# Secreted midbody remnants are a class of extracellular vesicles molecularly distinct from exosomes and microparticles

**DOI:** 10.1038/s42003-021-01882-z

**Published:** 2021-03-25

**Authors:** Alin Rai, David W. Greening, Rong Xu, Maoshan Chen, Wittaya Suwakulsiri, Richard J. Simpson

**Affiliations:** 1 Department of Biochemistry and Genetics, La Trobe Institute for Molecular Science (LIMS), La Trobe University, Melbourne, VIC 3086 Australia; 2 Baker Heart and Diabetes Institute, Melbourne, VIC 3004 Australia

**Keywords:** Extracellular signalling molecules, Proteomics

## Abstract

During the final stages of cell division, newly-formed daughter cells remain connected by a thin intercellular bridge containing the midbody (MB), a microtubule-rich organelle responsible for cytokinetic abscission. Following cell division the MB is asymmetrically inherited by one daughter cell where it persists as a midbody remnant (MB-R). Accumulating evidence shows MB-Rs are secreted (sMB-Rs) into the extracellular medium and engulfed by neighbouring non-sister cells. While much is known about intracellular MB-Rs, sMB-Rs are poorly understood. Here, we report the large-scale purification and biochemical characterisation of sMB-Rs released from colon cancer cells, including profiling of their proteome using mass spectrometry. We show sMB-Rs are an abundant class of membrane-encapsulated extracellular vesicle (200-600 nm) enriched in core cytokinetic proteins and molecularly distinct from exosomes and microparticles. Functional dissection of sMB-Rs demonstrated that they are engulfed by, and accumulate in, quiescent fibroblasts where they promote cellular transformation and an invasive phenotype.

## Abstract (web summary)

Rai et al. characterise the properties of secreted midbody remnants, showing they are distinct from exosomes and microvesicles. The authors also find that these vesicles are engulfed by cells and promote anchorage independent growth and invasive phenotypes in NIH3T3 fibroblasts.

## Introduction

At the terminal stage of cell division, the prospective daughter cells remain connected by a thin intercellular bridge containing the midbody (MB), a transient organelle responsible for mediating final abscission during cytokinesis^1–3^. The MB is then inherited by one of the newly-formed daughter cells where they perform non-mitotic functions^3–8^. While MB-Rs are degraded by autophagy^3^ they can also accumulate intracellularly and influence cell fate^8^. An alternative fate of MB-Rs involves extracellular secretion as sMB-Rs^9–11^. Over the past 30 years the prevailing view has been that sMB-Rs are degraded extracellularly^11^. However, recent evidence shows that sMB-Rs can also be engulfed by non-sister cells^8,12^. Because MB-Rs can influence cell signalling and cell fate in daughter cells^8–10,13,14^, it has been speculated that they might perturb cell signalling at distal sites^15^, however, this question remains largely unexplored.

Previously, we observed that MKLP1 (also known as KIF23 or ZEN-4)^16^, a component of the centraspindlin complex, co-purified with shed microvesicles (sMVs, also referred to as microparticles and ectosomes^17^) isolated from the cell culture medium of the human colorectal cancer (CRC) cell line LIM1863^18^. Because centraspindlin is a core component of MBs^16^ and MB-Rs^8^, we reasoned that MB-Rs might be released from cell lines into culture medium (CM) in sufficient quantities to permit their biochemical and functional characterisation. The focus of this paper is directed at the large-scale preparation of sMB-Rs that would allow us to undertake their biophysical and functional characterisation, to ask whether in vitro release of MKLP1 from LIM1863 CRC cells is a general phenomenon or a cancer-cell specific process, and whether MKLP1 is an indicative marker of sMB-Rs.

## Results and discussion

### Cancer cell-derived midbody remnants are shed into the extracellular space

**Fig. 1:**
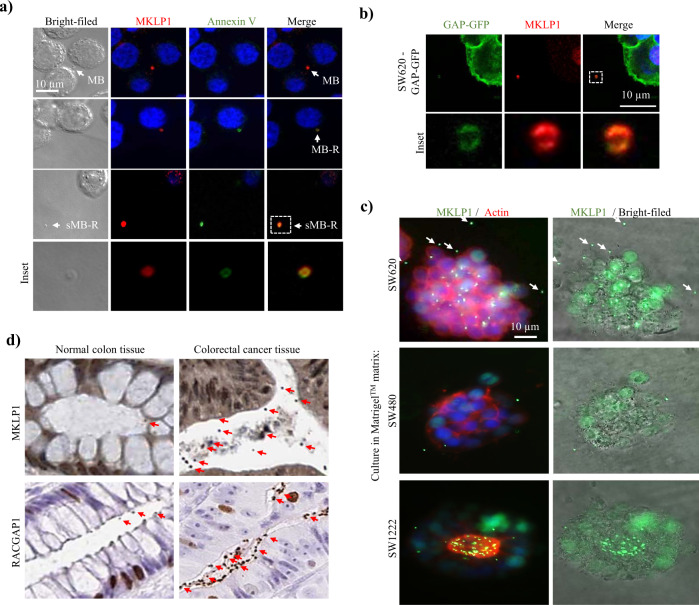
Secretion of cancer cells shed midbody remnants into extracellular space. **a** Immunofluorescence microscopy analysis of SW620 cells using anti-MKLP1 antibodies. SW620 cells cultured on glass coverslips were stained with annexin-V. Midbodies (MB) between prospective daughter cells do not stain with annexin-V (*top panel*). In contrast, midbody remnants (MB-R) associated with one of the cells stained with annexin V (*middle panel*). MB-Rs are also detected extracellularly as shed midbody remnants (sMB-Rs) (*bottom panel*). Inset panels represent enlarged images of sMB-Rs. Scale bar, 10 µm. **b** Immunofluorescence microscopy analysis of SW620-GAP-GFP cells using anti-MKLP1 antibodies. Nuclei (blue) were stained with Hoechst stain. Inset: higher magnification of GFP-tagged sMB-Rs in the extracellular space. Scale bar, 10 µm. **c** Immunofluorescence microscopy analysis of SW620, SW480 and SW1222 cells cultured in Matrigel^TM^ matrix using anti-MKLP1 antibodies (green) and Alexa Fluor 594 Phalloidin (red) to stain actin. Nuclei (blue) were stained with Hoechst stain. White arrow heads point show sMB-Rs. Scale bar, 10 µm. **d** Immunohistochemistry analysis of normal human colon tissue and colon cancer tissue (adenocarcinoma) using anti-MKLP1 and anti-RACGAP1 antibodies. Red arrows indicate anti-MKLP1 or anti-RACGAP1 staining extracellular sMB-Rs. Images obtained from Human Protein Atlas (http://www.proteinatlas.org/) with permission.

To determine whether sMB-Rs are secreted into the extracellular space, we first investigated whether MKLP1 could be used as a reliable marker for MB/MB-R detection. For this purpose we used CRC cells (SW620) grown in 2D culture. Fluorescent microscopy revealed that the MKLP1 antibody readily stained punctate structures nested in the middle of the intercellular bridge between dividing cells (revealed using beta-tubulin antibody) typical of MBs^1–3^ (Supplementary Fig. 1). Next we examined whether MKLP1 stained for MB-Rs. During the final stages of cytokinesis the inner leaflet phosphatidylserine (PS) of the MB membrane flips to the outer leaflet^19,20^ resulting in PS enrichment in the outer leaflet of the MB-R membrane. Importantly, PS on outer leaflet of sMB-Rs is required for their engulfment by cells^20^. In our study, fluorescent microscopy revealed that MBs localised between dividing SW620 cells did not stain with annexin V (Fig. 1a, *top panel*), whereas annexin V- and MKLP1-staining structures similar in size to MB-Rs associate with non-dividing cells. This observation is consistent with the presence of a MB-R inherited by one of the daughter cells post cytokinesis (Fig. 1a, *middle panel*). Moreover, co-staining of annexin V- and MKLP1 of MB-Rs was detected in the extracellular space (Fig. 1a, *bottom panel*) consistent with these particles being sMB-Rs. These anti-MKLP1-staining MB-R/sMB-R punctate structures were distinct from cellular debris as evidenced by lack of genomic DNA staining (Hoechst stain); their size range (~500 nm particle diameter) is consistent with previous report for sMB-Rs^9,10,19^. Collectively, these data show that MKLP1 can be used as a stereotypic marker for CRC cell MB/sMB-Rs. To provide additional proof that MB-Rs are secreted from cell lines into the extracellular space we generated fluorescently-labelled MB-Rs. For this purpose, we constructed SW620 cells stably-expressing plasma membrane-targeting GAP43 (1-20 a.a.)^21^ fused to GFP (SW620-GAP-GFP cells) (Supplementary Fig. 2). In Fig. 1b it can be seen that fluorescently-labelled/ MKLP1-positive MB-Rs are shed extracellularly.

MB-Rs have been reported to accumulate in small subpopulations of cancer cells and associate with stemness^8^. It can be seen in Supplementary Fig. 3a, b that while only a small population of SW620 and SW480 cells (0.9–1.6%) grown in culture accumulate MB-Rs, a large pool of non-cell associated MB-Rs are observed, a finding consistent with shedding of MB-Rs into the extracellular medium. This observation was corroborated using an antibody to the centraspindlin component RACGAP1 (Supplementary Fig. 3a). To rule out the possibility that MB-R shedding is an artefact of 2-D cell culturing, we prepared 3-D cultures of SW620 and SW480 cells in Matrigel^TM^ matrix (Fig. 1c) and tested for MB-R shedding. These data show that both SW620 and SW480 cells grown as spheroids also shed MB-Rs into their extracellular space (Fig. 1c, *upper and middle panels*).

Next, we set out to determine whether cancer cells shed MB-Rs in vivo. For this purpose, we established SW620-GAP-GFP cells as subcutaneous xenografts in mice. Immunohistochemical analysis of ectopic tumours from these mice using MKLP1 antibodies revealed GFP-tagged MB-Rs in the extracellular space (Supplementary Fig. 4). To determine whether sMB-Rs could be detected in human colon cancer tissues, we analysed MKLP1-or RACGAP1-antibody based immunohistochemical images of human colon cancer tissues publicly available from the Human Protein Atlas (http://www.proteinatlas.org/) (Fig. 1d, Supplementary Figs. 5 and 6). Strikingly, large pools of MKLP1- or RACGAP1-staining punctate structures were detectable in the extracellular space (lumen) of CRC tissues in greater abundance compared to non-disease colon tissue (Supplementary Fig. 7). Further, we also show that in stark contrast to non-polarised SW480- / SW620-spheroids that shed their sMB-Rs non-directionally (Fig. 1c), highly-polarised spheroids SW1222 cells (Fig. 1c, *lower panel*) or organoids cultured from mouse-derived intestinal crypts (Supplementary Fig. 8) shed their MB-Rs into the central lumen (stained by filamentous actin). These observations are consistent with the emerging role of MB-Rs in cell polarity^6,7^ and boost the notion that cancer cells actively shed MB-Rs into the extracellular space.

### sMB-Rs can be isolated from the culture medium of SW620 cells in high yield

**Fig. 2:**
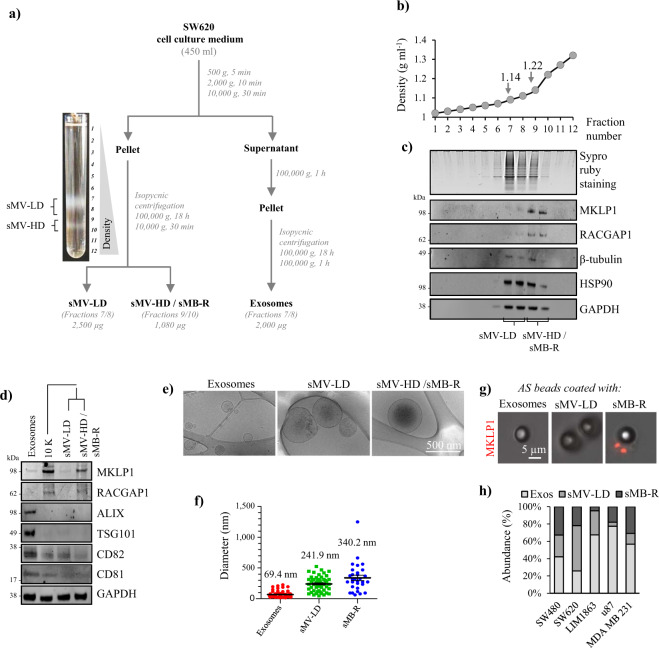
Isolation and characterisation of shed midbody remnants (sMB-Rs). **a** Experimental workflow for purification of sMB-Rs from SW620 cell culture medium (CM). CM was subjected to differential centrifugation to obtain crude sMVs (10,000 × *g* pellet) and exosomes (100,000 × *g* pellet) that were further fractionated using isopycnic density gradient centrifugation. Photographic image shows that crude sMVs (10,000 × *g* pellet) floated in two major fractions: low-density fractions 7/8 (sMV-LD) and high-density fractions 9/10 (sMV-HD/sMB-Rs). **b** The buoyant densities of twelve 1-mL fractions collected for each preparation were determined by absorbance at 244 nm using a molar extinction coefficient of 320 L g^−1^cm^−1^. **c** SDS-PAGE of 12 OptiPrep™ fractions. Protein quantitation was determined by SYPRO Ruby staining and western blot analysis performed using indicated antibodies. **d** Western blot analysis of SW620 cell-derived Exos, 10,000 × *g* EVs (crude sMVs), sMV-LD (fractions 7-8) and sMB-R (sMV-HD) (fractions 9-10) using indicated antibodies (*n* = 2 biological replicates). **e** Cryo-electron microscopic analysis of SW620 cell-derived Exos, sMV-LD and sMB-Rs. **f** Histogram represents the measurements of diameter of Exos, sMV-LD and sMB-R based on cryo-EM images. Data presented as mean ± s.e.m (standard error of mean). **g** Fluorescence microscopic analysis of Exos, sMVs-LD and sMB-Rs derived from SW620-GAP-GFP cells loaded onto aldehyde sulfate (AS) latex beads and immunostained with anti-MKLP1 antibodies (in red). **h** Bar plot showing protein yield (based on SYPRO Ruby protein quantitation) of Exos, sMVs-LD and sMB-R (sMVs-HD) secreted from five different cancer cell lines.

As a first step towards purifying sMB-Rs from CRC SW620 cell line culture medium it was important to establish which of the two major EV classes sMB-Rs belong to - exosomes or shed microvesicles/microparticles^17^. sMB-Rs from CRC SW620 cell line culture medium it was important to establish which of the two major EV classes sMB-Rs belong to - exosomes or shed microvesicles/microparticles^17^. EV classes differ in size range (exosomes typically 30–200 nm and sMVs, 50–1300 nm) and mode of biosynthesis (exosomes being of endosomal origin and sMVs forming by plasma membrane budding)^17^. Using a differential centrifugation strategy^22^, we first separated sMVs (which pellet at 10,000 × *g*) from exosomes which pellet at 100,000 × *g* (Fig. 2a) and showed sMB-Rs co-pellet with crude sMVs, but not exosomes as evidenced by western blot analysis using RACGAP1 antibody (Supplementary Fig. 9). Similar results were obtained using SW480 and LIM1863 cell lines (Supplementary Fig. 9). We next used an orthogonal step, isopycnic (iodixanol-density) centrifugation^23,24^, to further fractionate sMVs based on buoyant density (Fig. 2b, c). Two well-separated sMV fractions with distinct buoyant densities were found – low-density sMVs (sMV-LD fractions 7&8, 1.13–1.14 g ml^−1^) and high-density sMVs (sMV-HD fractions 9&10, 1.22–1.30 g ml^−1^) (Fig. 2a–c, Supplementary Fig. 10). Centraspindlin markers MKLP1 and RACGAP1 identified the sMV-HD fraction as highly enriched in sMB-Rs; this fraction was subjected to further biochemical and functional characterisation.

Both sMV-HD and -LD fractions displayed low or non-detectable levels of ALIX, TSG101, CD81 and CD82 and CD63 using Western Blotting (Fig. 2d, Supplementary Figs. 11 and 12), however, both ALIX and TSG101 were detected in sMB-R proteome data set (Supplementary Data 1). The yield of sMB-Rs (i.e., sMV-HD fraction) from 450 ml of SW620 cells grown in continuous culture (10 days, 3 Bioreactor Cell Line™ flasks) was 1080 µg protein (~67.986 × 10^6^ sMB-R particles). Cryo-electron microscopy revealed sMB-Rs (sMV-HD) with particle diameters in the range 200–600 nm partially overlapping with sMV-LD particles, but significantly larger than exosomes (30–200 nm) (Fig. 2e, f). This finding is comparable to particle size determinations obtained using nanoparticle-tracking analysis (Supplementary Fig. 13) and sMB-Rs (~300 nm) based on conventional electron microscopy^9^. The presence of MKLP1-positive sMB-Rs in sMV-HD fractions, but not sMV-LD or exosome fractions, was further validated by aldehyde sulfate latex bead capture/fluorescence microscopy (Fig. 2g). In RACGAP1 immunoprecipitation analysis of the sMV-HD fraction, MS-based proteomics identified MKLP1, an integral component of the MB centraspindlin complex of RACGAP1 and MKLP1/KIF23^16^ and other midbody components known to interact with RACGAP1, including PLK1, RHOA, CIT, KIF14 and KIF1A (Supplementary Data 2, Supplementary Fig. 14). We further showed that MB-R shedding by cancer cells is a widespread phenomenon, being observed for primary (e.g., SW480, LIM1863) as well as metastatic CRC cell lines (COLO 205 and T84) and the breast cancer cell line MDA MB 231 (Supplementary Figs. 15 and 16). Moreover, these data indicate that sMB-Rs (sMVs-HD) represent a significant portion of total secreted EVs (exosomes and sMVs-LD fractions) depending on cancer cell type (Fig. 2h).

### Protein profiling of shed midbody remnants

**Fig. 3:**
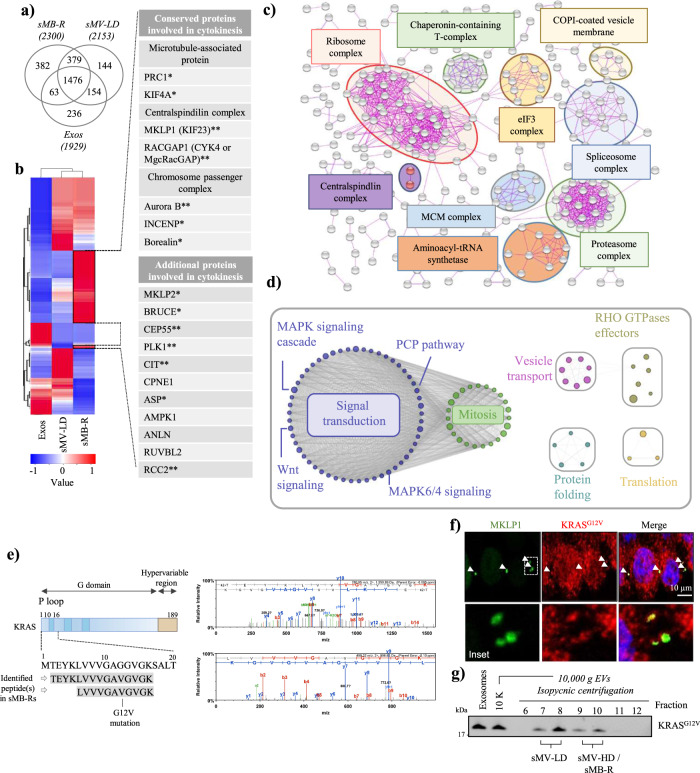
Proteome analysis of shed midbody remnants. **a** Venn diagram of proteins identified in SW620 cell-derived sMB-R (sMV-HD), sMV-LD and Exos. **b** Heatmap illustration of proteins identified in sMB-R (sMV-HD), sMV-LD and Exos. Proteins present in higher abundance in sMB-R (red) as compared to sMV-LD and Exos include conserved cytokinetic proteins as well as additional cytokinetic proteins. *Proteins uniquely identified in sMB-Rs. **Proteins enriched (fold change >2) in sMB-R compared to sMV-LD and Exos. **c** STRING-based protein-protein interaction network analysis of 928 enriched proteins in sMB-Rs (sMV-HD) compared to sMV-LD and Exos. The interactions were “evidence”-based, with “experiments” as active interaction source and interaction threshold set at 0.900 (highest confidence). Disconnected nodes in the network are hidden. Proteins identified under biological processes or molecular processes (Gene Ontology) are indicated. Centralspindlin complex components (RACGAP1 and KIF23/MKLP1) are also indicated. **d** EnrichmentMap of Reactome pathways enriched in 456 proteins commonly identified in SW620 cell-derived sMB-R proteome (2300 proteins) with the proteome of MB-Rs shed by Hela cells reported recently by Peterman et al. 2019^20^. **e** Mass spectrometry-based identification of KRAS peptides (UniProtKB ID RASK_HUMAN) in sMB-Rs. Two peptides (TEYKLVVVGAGGVGK and LVVVGAGGVGK) spanning Gly-12/ Val-12 substitution in KRAS protein. Peptide spectral profiles are displayed on the right. **f** Immunofluorescence microscopy of SW620 cells using anti-MKLP1 and anti-KRAS^G12V^ antibodies. Nuclei (blue) were stained with Hoechst stain. White arrows indicate the position of MB and MB-Rs. Inset represents higher magnification. Scale bar, 10 µm. **g** Western blot analysis of exosomes, crude 10,000 x *g* sMVs, and isopycnic (iodixanol-density) gradient centrifugation fractions of sMV-LD and -HD/sMB-Rs using anti-KRAS^G12V^ antibody.

Next, we performed a comparative proteome analysis of SW620 cell-derived sMB-Rs (sMV-HD), sMV-LD and exosomes using a label-free quantitative mass spectrometry (MS) approach^18^. A total of 2300 proteins were identified in sMB-Rs, 2153 in sMVs-LD, and 1929 in exosomes (Supplementary Data 1) and 382, 144 and 236 proteins, respectively are uniquely identified (Venn diagram, Fig. 3a) indicating that these three vesicle types are molecularly distinct from one another.

The relative abundance of proteins in each EV subtype, based on normalised spectral counts, is shown in the heatmap (Fig. 3b). Notably, proteins associated with cytokinesis such as microtubule-bundling proteins^25^, the centraspindlin complex^16^ and chromosomal passenger complex^26^ are selectively enriched in sMB-Rs (the sMV-HDs fraction), but not in sMV-LD and exosome fractions. These cytokinesis-signature proteins found exclusively in sMB-Rs boost our argument that sMB-Rs represent a new category of EV, hitherto undescribed in the EV literature.

Next, using the STRING database (version 10.5) we identified 982 high-abundance SW620 cellular proteins in sMB-Rs, compared to exosomes and sMV-LD (Supplementary Data 3 (highlighted in red in heatmap, Fig. 3b)). Using this list, we constructed a protein-protein interaction network for sMB-Rs proteins (Fig. 3c). GO analysis identified protein clusters implicated in biological processes such as RNA regulation (e.g., “ribosome”, “aminoacyl-tRNA synthase”, “eIF3 complex” and “spliceosome”), Protein degradation (e.g., “proteasome complex”), and Vesicle transport (e.g., “COPI-coated vesicle membrane”) (Fig. 3c). Strikingly, these clusters include proteins important in biological processes such as translation^27,28^, protein degradation^29–32^, and vesicle transport^1,2,33^ - processes reported to be tightly-regulated in MBs and critical for faithful cytokinesis^1,2,27–36^.

Furthermore, 32/982 of high-abundance sMB-Rs proteins are listed in the MiCroKITS-v4.0 database of proteins ‘experimentally-verified to temporally and spatially localise to midbody, centromere, kinetochore, telomere or spindle structures during cell division’^37^ (http://microkit.biocuckoo.org, Supplementary Data 4).

To further address the functionality of sMB-R proteins, we conducted a gene-annotation enrichment and pathway analysis (DAVID^38^) (version 6.8) using the Gene Ontology (GO) and Kyoto Encyclopaedia of Genes and Genomes (KEGG) databases (Supplementary Data 5). This analysis revealed 207 proteins in sMB-Rs involved in regulation of “signal transduction”; amongst these ‘MAPK signalling”, “Ras signalling pathway” and “Pathways in cancer” are preeminent (Supplementary Fig. 17).

We next compared SW620 cell derived sMB-R proteome (2300 proteins) with the proteome of MB-Rs shed by Hela cells reported recently by Peterman et al.^20^. A total of 456 proteins were commonly identified (Supplementary Fig. 18, listed in Supplementary Data 6). We next performed Reactome pathways analysis on 456 common proteins (Supplementary Data 7). EnrichmentMap of the top 100 (*q*-value < 0.0001) pathways (Fig. 3d) revealed significant enrichment of mitotic processes (39 proteins including centralspindlin complex proteins MKLP1 and RACGAP1), signal transduction pathways (including 38 MAPK signalling pathway proteins such as MEK1/2/3, IQGAP1, PAK1/2, RAC1, SEPTINN7, SPTAN1, TLN1, XPO1, YWHAB), and RHO GTPase effectors (IQGAP1, IQGAP3, ITGB1, PAK1/2, RAC1/2, RHOA/G, XPO1, YWHAB/E/H/Q/Z)). A complete list of proteins involved in these pathways is given in Supplementary Data 8. We validated the expression of one such protein (KRAS^G12V^ oncoprotein (Fig. 3e)) in SW620 cell-derived sMB-Rs by mass spectrometric analysis as well as by fluorescence microscopy and western blotting (Fig. 3f, g).

### Shed midbody remnants are taken up by fibroblasts

**Fig. 4:**
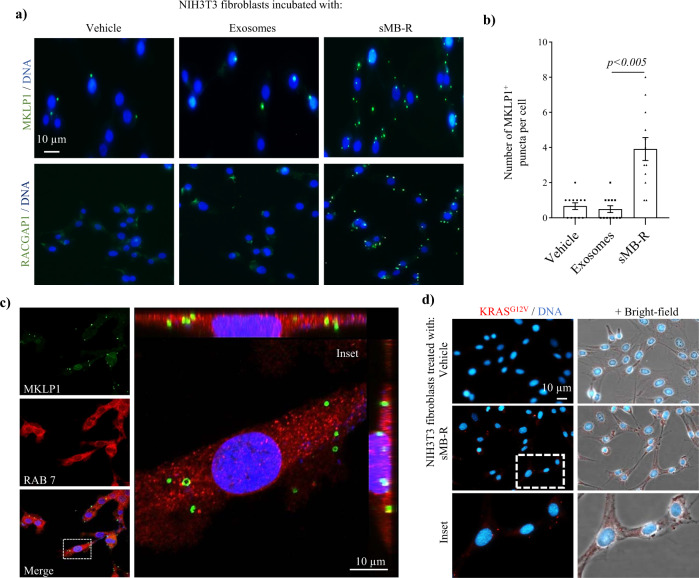
Internalisation of cancer cell-derived shed midbody remnants by fibroblasts. **a** Uptake of sMB-Rs by fibroblasts. Fluorescence microscopy analysis of NIH3T3 fibroblasts incubated with/without SW620 cell-derived sMB-Rs or Exos (50 µg ml^−1^) for 2 h using anti-MKLP1 and anit-RACGAP1 antibodies. **b** Uptake and accumulation of sMB-Rs in NIH3T3 fibroblasts was quantified by counting MKLP1^+^ puncta per cell; data represented as mean ± s.e.m. Nuclei (blue) were stained with Hoechst. Scale bar, 10 µm. **c** Internalisation of sMB-Rs by fibroblasts. Confocal microscopy of NIH3T3 fibroblasts incubated with sMB-Rs using anti-MKLP1 (in green) and anti-RAB7 (in red) antibodies. Confocal microscopy analysis along Z-axis (inset) reveal internalisation of sMB-Rs following uptake. Scale bar, 10 µm. **d** Intercellular transfer of sMB-R KRAS^G12V^ into NIH3T3 cells. Fluorescence microscopy of NIH3T3 fibroblasts incubated with SW620 cell-derived sMB-Rs (5 µg) for 2 h using anti-KRAS^G12V^ antibodies. Nuclei were stained with Hoechst stain (blue). Right panel represents fluorescence signals from left panel overlaid onto bright-field images. Inset represents enlarged image. Scale bar, 10 µm.

To address whether sMB-Rs from cancer cells can influence non-cancer cells, like exosomes and microparticles, we treated NIH3T3 fibroblasts with purified sMB-Rs for 2 h and then used MLKP1 and RACGAP1 antibodies to evaluate vesicle uptake (Fig. 4a). Compared to untreated fibroblasts, sMB-R-treated fibroblasts displayed an approximate 4-fold increase in uptake and accumulation of MKLP1/RACGAP1-positive sMB-Rs (in green, up to eight sMB-R green puncta per recipient cell) (Fig. 4a, b). Uptake of sMB-Rs was evident within 1 h (Supplementary Fig. 19). Confocal microscopy revealed that sMB-Rs were internalised by NIH3T3 fibroblasts (Fig. 4c) and can deliver their protein cargo (Fig. 4d).

We envision that several cargo proteins in sMB-Rs collectively signal in recipient cells. These include MEK1/2/3, IQGAP1, PAK1/2, RAC1, SEPTINN7, SPTAN1, TLN1, XPO1 and YWHAB, which are effectors of various signalling pathways. Additionally, in our proteomic data set we also detect FGF3 and its receptor FGFR4. Because sMB-Rs released into the extracellular space during symmetric abscission contain the membrane envelope that originates from the plasma membrane of the parent cell, it is conceivable that MB-Rs might deliver functional FGF3/FGFR4 signalling complex to the recipient cells and activate its downstream signalling pathways^39^. It is well documented that soluble secreted signalling molecules bound to their cognate receptors can be loaded onto the EV-surface (for example, TFGβ-1^40^). Non-specific binding of particles on EV surfaces has also been observed in physiological conditions such as binding of lipoprotein particles to blood EVs^41^. Whether specific or non-specific binding of factors on vesicular surfaces is of physiological significance remains an open question. Thus, we anticipate that several players collectively signal upon sMB-R uptake in recipient cells. In addition, although sMB-Rs are internalised by fibroblasts, downstream signalling could be mediated by engagement of surface receptors^20^.

### SW620 cell-derived sMB-Rs promote anchorage independent and invasive phenotype in fibroblasts

**Fig. 5:**
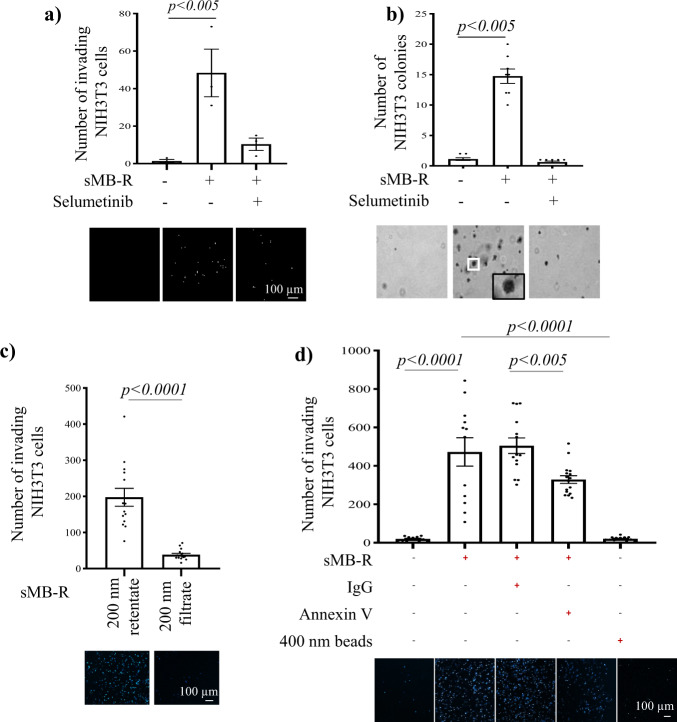
Cancer cell-derived shed midbody remnants induce invasive/transformed phenotype in NIH3T3 fibroblasts. **a** sMB-Rs confer invasive capability in NIH3T3 fibroblasts. Transwell-Matrigel^TM^ invasion assay of NIH3T3 cells treated with SW620-derived sMB-Rs NIH3T3 fibroblasts were incubated with SW620 cell-derived sMB-Rs (30 µg ml^−1^) for 2 h and overlaid onto Matrigel^TM^ matrix coated inserts on a Transwell invasion assay and the number of invading cells quantified. Data represented as mean ± s.e.m. **b** sMB-Rs confer anchorage-independent growth capability in NIH3T3 fibroblasts. Soft agar colony formation assay of NIH3T3 treated with SW620-derived sMB-Rs. NIH3T3 fibroblasts were incubated with SW620 cell-derived sMB-Rs (200 µg ml^−1^) for 2 h and then grown in 0.6% soft-agar suspension in a soft agar colony formation assay. Data represented as mean ± s.e.m. **c** Transwell-Matrigel^TM^ invasion assay of NIH3T3 cells treated sMB-Rs obtained using size-based purification (0.22 μm pore size filter). The filtrate (flowthrough that contains small EVs (30–150 nm) and soluble protein contaminants) and the retentate (containing larger sMB-Rs) were used to assess fibroblast invasion. Data represented as mean ± s.e.m. Lower panel: fluorescence microscopy images of nuclei (stained with Hoechst) of invasive fibroblasts. **d** Transwell-Matrigel^TM^ invasion assay of fibroblasts treated with by sMB-R fraction depleted of sMB-Rs using biotin-Annexin V or 400 nm Polystyrene latex bead. Data represented as mean ± s.e.m. Lower panel: fluorescence microscopy images of nuclei (stained with Hoechst) of invasive fibroblasts.

Because cancer EVs play an important role in cellular transformation, such as acquisition of invasive phenotype and anchorage independent cell growth capacity^42^, we reasoned that SW620-derived sMB-R uptake by NIH3T3 fibroblasts might promote cell invasion and anchorage-independent growth capacity. To test whether sMB-Rs promote cell invasion, NIH3T3 fibroblasts were incubated with sMB-Rs (30 µg ml^−1^) for 2 h, overlaid onto Matrigel^TM^ matrix coated inserts on a Transwell invasion assay plate, and the number of invading cells quantified (Fig. 5a). In contrast to control (untreated) fibroblasts that failed to invade, sMB-R-treated fibroblasts displayed significantly higher invasive capacity (>14-fold increase, *p* < 0.005, Fig. 5a); this invasive capacity was attenuated by pre-treatment of NIH3T3 fibroblasts with MEK inhibitor selumetinib (AZD6244). Next, to test whether sMB-Rs promote anchorage-independent growth, NIH3T3 fibroblasts were incubated with sMB-Rs (30 µg ml^−1^) for 2 h and grown in 0.6% soft-agar suspension in a soft agar colony formation assay (Fig. 5b). Compared to control fibroblasts treated with vehicle alone that failed to form colonies, sMB-R-treated fibroblasts formed significantly greater numbers of colonies (>14-fold increase, *p* < 0.005) on soft agar, which was attenuated by pre-treatment of NIH3T3 fibroblasts with selumetinib. This fibroblast cell transforming capacity of sMB-Rs is comparable to that of exosomes and sMVs-LD (Supplementary Fig. 20). Furthermore, we subjected purified sMB-Rs to size-based filtration (220 nm) to remove any remaining exosomes (Fig. 5c). The pro-invasive signalling capacity on fibroblasts by purified sMB-Rs was solely observed in the retentate that contains larger sMB-Rs *versus* the flowthrough, which contains residual exosomes and soluble secretome. We also show using 400 nm polystyrene latex beads that the acquired invasive capacity is not due to mere exposure to particles (Fig. 5d). Additionally, depletion of sMB-Rs using Annexin V resulted in ~30% reduction in the invasive phenotype of NIH3T3 fibroblasts (Fig. 5d). Although Annexin V has been shown to promote invasion in cancer cells^43,44^, Peterman et al.^20^ showed that PS on the outer leaflet of sMB-Rs is required for their engulfment by cells, thus any residual Annexin V carrying over to the invasion assay, albeit in very low amounts, would potentially reduce sMB-R-uptake by fibroblasts and thereby reduce their invasive capacity.

The mechanism by which sMB-Rs exert long-term effect is currently not understood. Similar to our findings, one-time treatment of MDA-MB-231 cells with sMB-Rs from Hela cells resulted in enhanced soft agar colony forming capacity (as evident from larger colonies over 14 days)^20^. Further, Peterman et al. showed that MB-Rs taken up by recipient cells were still present after 48 h post-feeding, potentially allowing for continuous signalling through at least two different pathways: αVβ3-FAK-Src and EGF-EGFR. Similar observations has also been made in the EV field where a single stimulation of U373 cells with EGFRvIII-containing EV caused a 2-fold increase in anchorage-independent soft-agar colony formation of U373 cells (over a period of 3 weeks), whereas exposure to the equivalent amount of microvesicles devoid of EGFRvIII resulted in no significant increase in colony forming capacity^45^.

**Fig. 6:**
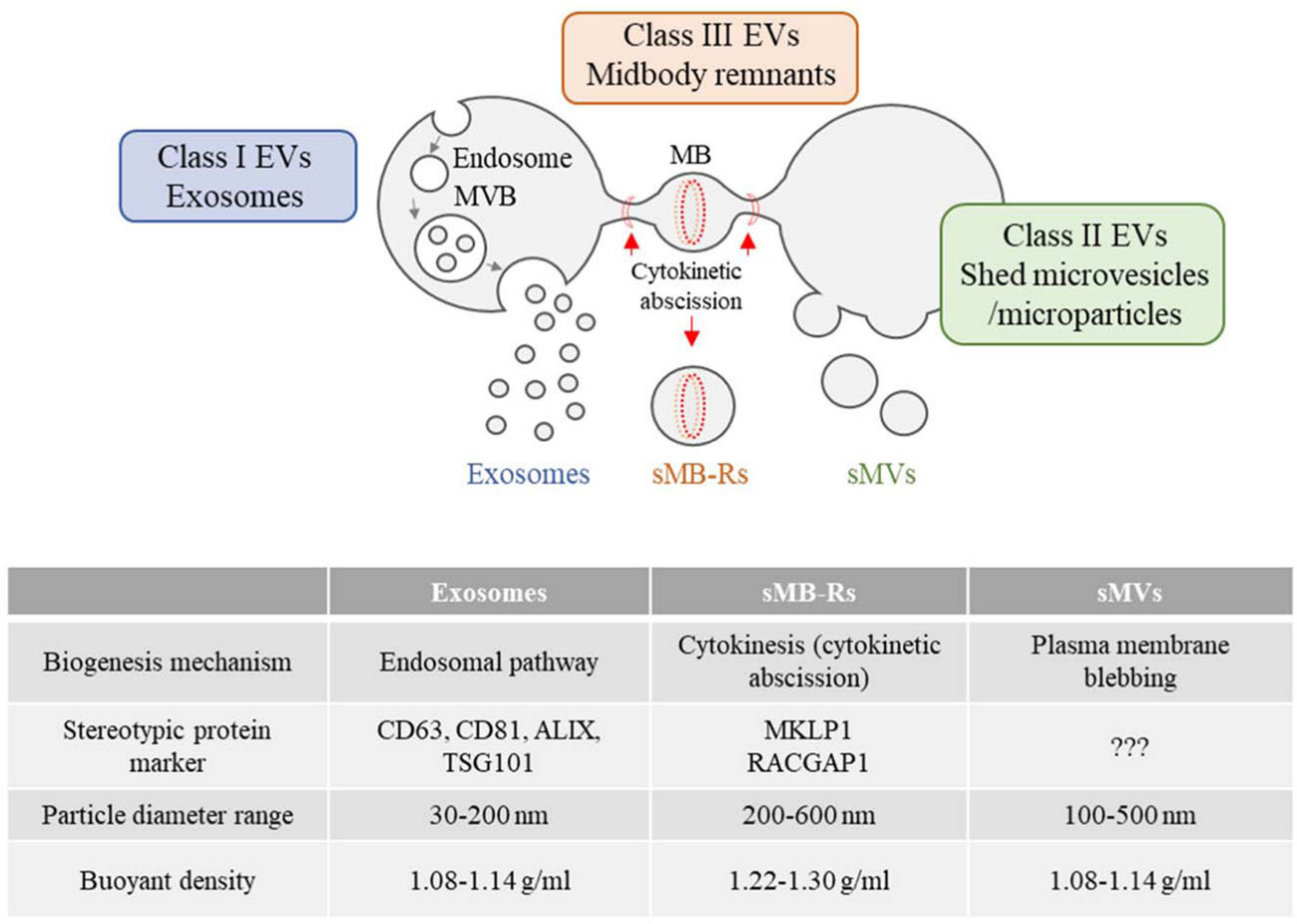
Schematic illustration of three distinct classes of extracellular vesicles – exosomes, shed microvesicles/microparticles and shed midbody remnants (sMB-Rs). Exosomes (class I EVs) are of endosomal origin (formed by invagination of multivesicular bodies (MVB)), shed microvesicles/ microparticles (class II extracellular vesicles) are formed via direct outward blebbing of plasma membrane and midbody remnants (MB-Rs) (class III extracellular vesicles) are generated by cytokinetic abscission of the interconnecting bridge between dividing daughter cells (post completion of cytokinesis). Current understanding of biophysical properties and stereotypic marker proteins for exosomes, sMVs (microparticles) and sMB-Rs are listed in the table. Centraspindlin complex proteins MKLP1 and RACGAP1 enable distinction of sMB-Rs from exosomes and sMVs.

These findings suggest that upon uptake of sMB-Rs, initial signalling in recipient cells is sufficient to support or enhance a phenotype, in this case anchorage independent growth, for up to 10–14 days. Alternatively, MB-Rs may persist longer than 48 h to continually signal. In this regard, several studies have shown that MB-Rs persist in cells for extended periods. For example, accumulation of asymmetrically inherited MB remnants (up to 20 MB-Rs per cell) was shown to persist in stem cells and cancer cells^8^. Exogenously supplemented sMB-Rs have also been shown to persist for up to 48 h in cells following uptake^20^. While, phagocytosed particles are rapidly degraded within 2–5 h by fusing with lysosomes, internalised sMB-Rs can persist within actin-coated endosomes and thereby evade lysosomal degradation^8,20^. In our study, we show that sMB-Rs can persist in recipient cells for up to 48 h (Supplementary Fig. 21). However, it is conceivable that in vivo, continuous exposure to sMB-Rs might be required to support reprogrammed phenotype in recipient cells, as is the case of exosomes within the TME^45^. Although EVs have been previously shown to transfer mutated proteins that function in recipient cells, whether sMB-Rs deliver functional mutant KRAS warrants future investigation. On the other hand, sMB-Rs might be able to mediate epigenetic reprogramming of cells in a manner similar to exosomes^46^. In conclusion, characterising the biochemical properties of sMB-Rs is an indispensable first step towards understanding their underlying functionality. In this work we show that colon cancer-derived sMB-Rs can be isolated in high yield from the culture medium of SW620 cells grown in continuous culture tanks. Using a combination of differential centrifugation (10,000 × *g*) and OptiPrep™ (iodixanol) density gradient centrifugation ~1 mg of highly-purified sMB-Rs (based on protein concentration) exhibiting a range of particle diameter (200–600 nm) and buoyant density (in the range 1.22–1.30 g ml^−1^) were obtained. GeLC-MS/MS analysis shows that sMB-Rs contain “cytokinesis signature proteins” (microtubule-bundling proteins, the centraspindlin complex (MKLP1/KIF23and RACGAP1) and chromosomal passenger complex proteins) not seen in exosomes and sMVs/microparticles. Functional studies show that sMB-Rs, like exosomes and low-density sMVs (buoyant density 1.08–1.14 g ml^−1^) can be taken up and accumulate in quiescent fibroblasts where they promote cellular transformation and a pro-invasive phenotype^47,48^. Collectively, our findings show for the first time that sMB-Rs represent a third major class of EV molecularly distinct from exosomes and shed microvesicles/microparticles (Fig. 6). Our findings provide significant insights into sMB-R biology, suggesting this class of circulating EV may not merely constitute remnants of cytokinesis, but might also possess an unexpected role in cancer biology.

## Methods

### Cell lines and cell culture

SW620 (CCL-227, ATCC), SW480 (CCL-228, ATCC), LIM1863 cells^49^ (Ludwig Institute for Cancer Research, Melbourne), COLO 205 (CCL-222, ATCC), T84 (CCL-248, ATCC), SW1463 (CCL-234, ATCC), SW1222 (12022910, CellBank Australia), LIM2405 (12062003, Sigma Aldrich) and LIM2408 (The Ludwig Institute for Cancer Research, Melbourne) cells were cultured in RPMI-1640 (Life Technologies). NIH3T3 fibroblasts (CRL-1658, ATCC), MDA MB 231 (HTB-26, ATCC), U87 (HTB-14, ATCC), HCT15 (CCL-225, ATCC), HCT116 (CCL-247, ATCC), HT29 (HTB-38, ATCC) and HCA7 (06061902, CellBank Australia) were cultured in DMEM (Life Technologies). Media were supplemented with 10% (v/v) Foetal Bovine Serum (FBS) (Life Technologies) and 1% (v/v) Penicillin Streptomycin (Pen/Strep, Life Technologies) and maintained at 37 °C with 10% CO_2_. Cells were routinely passaged using trypsin-EDTA (Gibco).

### Isolation of exosomes, sMV-LD and sMV-HD/sMB-Rs

Cells (SW620, SW480, MDA MB 231 and U87) were cultured in CELLine AD-1000 Bioreactor classic flasks (Integra Biosciences)^50^. SW620 or SW480 cells (3 × 10^7^) in 15 ml of RPMI media (supplemented with EV-depleted 10% FBS and 1% Pen/Strep), or MDA MB 231 or U87 cells (3 × 10^7^) in 15 ml of DMEM medium (supplemented with EV-depleted 10% FBS, 1% Pen/Strep) were added to the lower cell-cultivation chamber. The upper nutrient supply chamber contained 500 ml RPMI or DMEM (5% FBS, 1% Pen/Strep) that was replaced every 4 days. Cells in the lower cell cultivation chamber were allowed to adhere for 72 h at 37 °C with 10% CO_2_. Thereafter, the lower chamber was gently washed with serum-free RMPI or DMEM medium. For SW620 or SW480 cells, 15 ml of RPMI media (supplemented with 0.5% (v/v) insulin transferrin selenium (Invitrogen) and 1% Pen/Strep) was added. For MDA MB 231 or U87 cells, 15 ml of DMEM medium (supplemented with EV-depleted 10% FBS, 1% Pen/Strep) were added to the lower cell-cultivation chamber. Thereafter, culture medium (CM) in the cell cultivation chamber was replaced each day. CM was sequentially centrifuged at 500 × *g* for 5 min (4 °C) and 2000 × *g* for 10 min (4 °C). The resultant supernatant was centrifuged at 10,000 × *g* (30 min) to pellet crude sMVs. The supernatant was then centrifuged at 100,000 × *g* (1 h) to pellet crude exosomes. Crude sMVs and crude exosomes were resuspended in 500 µl PBS and subjected to isopycnic (iodixanol-density) ultracentrifugation^24,51^. Briefly, a discontinuous gradient of OptiPrep^TM^ (iodixanol solution) was prepared by layering 40% (3 ml), 20% (3 ml), 10% (3 ml) and 5% (2.5 ml) of iodixanol solution in a 14 × 89 mm polyallomer tube (Beckman Coulter). Dilutions of iodixanol solution were made in 0.25 M sucrose/10 mM Tris (pH 7.5) solution. Crude sMVs and crude exosomes (in 500 µl PBS) were overlaid and subjected to centrifugation at 100,000 × *g* for 18 h (4 °C). Next, twelve 1-ml fractions were collected, diluted in PBS (2 ml) and centrifuged at 100,000 × *g* for 1 h. The supernatant was discarded and pellets were further washed in PBS (500 µl) with final resuspension made in 100 µl of PBS and stored at −80 °C until further use. Human colon carcinoma LIM1863 cells were cultured in T175 flasks in RPMI medium supplemented with 0.5% (v/v) insulin transferrin selenium and 1% Pen/Strep and CM harvested as previously described^18^ and subjected to EV isolation strategy as described above.

Size-based purification of sMB-Rs was performed using Ultrafree-CL Centrifugal Filter (Millipore, 0.22 µm pore size, hydrophilic PVDF). Briefly, purified sMB-Rs (150 µg in 600 µl PBS) was filtered using Ultrafree-CL Centrifugal Filter at 1000 × *g* (5 min, 4 °C). The filtrate (flowthrough that contains small EVs (30–200 nm) and soluble protein contaminants) and the retentate were then centrifuged at 10,000 × *g* (30 min, 4 °C). The pellets were resuspended in 150 µl PBS used for Transwell-Matrigel^TM^ invasion assay (see below) at 1:1 (Fig. 5c).

### Protein quantification and western blotting

Protein samples were quantified by 1D SDS-PAGE/SYPRO Ruby protein staining-based densitometry^18^. Western blotting was performed on protein samples (10–20 µg) as previously described^18^. Rabbit antibodies raised against GAPDH (Cell Signalling), β-tubulin (Cell Signalling), KRAS^G12V^ mutant specific (Cell Signalling), RAB7 (Abcam) and GFP (Abcam) were used. Mouse antibodies against MKLP1 (Santa Cruz), RACGAP1 (Santa Cruz), ALIX (BD Biosciences), TSG101 (BD Biosciences), CD63 (Santa Cruz), CD81 (Santa Cruz), RAB2A (Thermo Fisher), FLOT1 (BD Biosciences), α-actinin (Abcam), CD9 (Santa Cruz) and HSP90 (BD Biosciences) were used. Secondary antibodies used were IRDye 800 goat anti-mouse IgG or IRDye 700 goat anti-rabbit IgG (1:15000, LI-COR Biosciences).

### Cryo-EM and NTA

Cryo-electron microscopy (Tecnai G2 F30) on EVs (2 µg) was performed as described^18^. Vesicle particle size was determined using a NanoSight NS300, Nanoparticle tracking analysis (NTA) (Malvern) system fitted with a NS300 flow-cell top plate with a 405 nm laser. Samples (1 µg µl^−1^) were diluted in 500 µl PBS (1:10,000) and injected using 1 ml syringes (BD Biosciences) (detection threshold = 10, flowrate = 50, temperature = 25 °C). Each analysis consisted of 60 s video captures. Data was analysed using NTA software 3.0 (Malvern).

### Immunofluorescence assay

Immunofluorescence was performed, as previously described^47^. Briefly, cells were cultured on Nunc® Lab-Tek® Chamber Slide™ (Sigma-Aldrich) system to 60–80% confluency. Cells were washed, fixed (4% formaldehyde for 10 min), permeabilized (0.2% (v/v) Triton X-100 in TTBS, 5 min) and blocked (3% (w/v) bovine serum albumin (BSA, Sigma) in TTBS (0.2% (v/v) Triton X-100) (blocking solution) for 30 min at room temperature. Cells were then incubated with primary antibodies (1:100) (mouse anti-MKLP1 (Santa Cruz Biotechnology), mouse anti-RACGAP1 (Santa Cruz), rabbit anti-KRAS^G12V^ mutant specific (Cell Signalling), rabbit anti-RAB7 (Abcam) and rabbit anti-β-tubulin (Cell Signalling) in blocking solution for 1 h at room temperature. Cells were washed and incubated with secondary antibodies (1:200) (Alexa Fluor 488-conjugated goat anti-mouse IgG or Alexa Fluor 568-conjugated goat anti-rabbit IgG (Invitrogen) in blocking solution for 20 min at room temperature (in the dark). Cells were washed 3× in TTBS. Where indicated, nuclei were stained with Hoechst stain (10 µg ml^−1^) for 1 min and actin labelled with Alexa Fluor 555 Phalloidin (Cell Signalling). Cells were imaged using a Zeiss AxioObserver Z1 microscope (Zeiss) or Zeiss Confocal LSM 780 PicoQuant FLIM (Zeiss) and images were analysed using Zen 2011 (Blue edition, Zeiss). For annexin V staining, live cells were labelled with Annexin V, Alexa Fluor™ 488 conjugate (ThermoFisher) according to the manufacturer’s instructions. Briefly, cells were washed in cold PBS, and incubated with annexin binding buffer (5 µl of the annexin V conjugate in 100 µl of 10 mM HEPES, 140 mM NaCl, and 2.5 mM CaCl_2_, pH 7.4) for 15 min at room temperature. Cells were washed with PBS, followed by fixation and immunofluorescence assay as above.

For 3-D culture, 500 cells were mixed with 50 µl Growth Factor-Reduced Matrigel^TM^ matrix (Corning) and overlaid onto a Nunc® Lab-Tek® Chamber Slide™ system. The matrix was allowed to polymerise at 37 °C for 1 h and gently overlaid with growth medium. After 4–8 days, 3-D cultures were fixed with 2% aqueous formaldehyde and subjected to immunofluorescence assay.

Animal experiments were performed in accordance with La Trobe University Ethics committee guidelines. SW620 GAP GFP cells (1 × 10^6^ cells/site) were subcutaneously injected into both inguinal regions of two NOD/SCID male mice to establish a total of four tumour xenografts. After 4 weeks, mice were killed, and tumours were excised, fixed in 4% aqueous formaldehyde, incubated in 20% sucrose solution for 48 h, embedded in optimum cutting temperature solution (Tissue-Tek®) and frozen (using isopentane). Sections (20 µm) were then subjected to immunofluorescence assay using mouse anti-MKLP1 antibody (1:100).

Isolation and culturing of intestinal crypts (as organoids) from small intestine or colon of C57BL/6 mice was performed using Gentle Cell Dissociation Reagent (STEMCELL^TM^ Technologies) and IntestiCult™ Organoid Growth Medium (Mouse) (STEMCELL^TM^ Technologies) as per manufacturer’s instructions. Organoids were cultured in Growth Factor-Reduced Matrigel^TM^ matrix for 7–10 days, fixed with 2% aqueous formaldehyde and subjected to immunofluorescence assay.

To quantify sMB-R particle number, 10 µg of sMB-Rs were subjected to immunofluorescence labelling as described above, using either mouse anti-MKLP1 (1:100, Santa Cruz Biotechnology) or mouse IgG isotype matched antibody (Abcam), and probed with Alexa Fluor 488-conjugated goat anti-mouse IgG (1:200). Labelled sMB-Rs were embedded in Matrigel^TM^, and MKLP1 + particles imaged using Zeiss AxioObserver Z1 microscope and numbers quantified using Image J software v1.49e.

### RACGAP1 immunoprecipitation assay

Dynabeads^TM^ Protein G (Life Technologies) (10 µl) were conjugated with 1 µg RACGAP1 antibody (Santa Cruz) or mouse IgG isotype matched antibody (BD Biosciences) for 15 min at room temperature under continuous rotation. Antibody-bead conjugates were collected using a magnet. Next, sMB-Rs (200 µg) were solubilized in 0.5% TX-100-PBS (supplemented with Complete^TM^ EDTA-free Protease Inhibitor Cocktail (Roche) and PhosSTOP^TM^ Phosphatase inhibitor (Roche)) on ice for 30 min. Samples were centrifuged at 5000 × *g* for 1 min. The resultant supernatant was then incubated with antibody-conjugated Dynabeads^TM^ Protein G for 2 h at 4 °C. Beads were washed 3× with 0.2% TX-100-PBS and proteins eluted in SDS sample buffer and analysed by GeLC-MS/MS.

### EV loading on aldehyde/sulphate latex beads

EVs (30 µg) were incubated with 1 µl aldehyde/sulphate latex beads (Invitrogen) (total volume, 500 µl PBS) for 15 min (room temperature) with continuous rotation. The reaction was stopped using 100 mM glycine and 2% BSA in PBS for 30 min (room temperature) with continuous rotation. Beads were centrifuged at 5000 × *g* (2 min), washed again with 300 µl PBS, permeabilized (0.2% TX-100 in PBS for 5 min), blocked (0.2% TX-100, 10% BSA in PBS for 10 min), washed and incubated with anti-MKLP1 antibody (1:50 dilution in 0.2% TX-100/2% BSA in PBS for 1 h) at room temperature with continuous rotation. Beads were washed 3× and incubated with Alexa Fluor 488-conjugated goat anti-mouse IgG (Invitrogen) at 1:100 dilution in 0.2% TX-100/2% BSA in PBS for 20 min under continuous rotation. Beads were washed 3×, resuspended in PBS and imaged using Zeiss AxioObserver Z1 microscope (Zeiss).

### GeLC-MS/MS and data analysis

Proteomic experiments were performed in two independent biological replicates (with technical duplicates) using GeLC-MS/MS for each sample (sMB-Rs, exosomes, sMV-LD) as described previously^18^. Raw data was processed using Proteome Discoverer (v2.1, Thermo Fischer Scientific) and searched with Mascot (Matrix Science, London, UK; v2.5), Sequest (Thermo Fisher Scientific, San Jose, CA, v1.4.0.288), and X! Tandem (v2010.12.01.1) against the UniProt Human database comprising 71,785 entries. Data was searched with a parent tolerance of 10 ppm, fragment tolerance of 0.5 Da and minimum peptide length 7, with FDR 1% at the peptide and protein levels. Peptide spectral matches were validated using Percolator based on q-values at a 1% false discovery rate (FDR)^52^. Scaffold Q+/Q + S (Proteome Software Inc., Portland, OR, v4.8.7) was employed to validate MS/MS-based peptide and protein identifications from database searching^53^. Initial peptide identifications were accepted if they could be established at greater than 95% probability as specified by the Peptide Prophet algorithm. Protein identifications were accepted, if they reached greater than 99% probability and contained at least two identified unique peptides. Protein probabilities were assigned using Protein Prophet^54^. The relative abundance of a protein within a sample was determined using normalised spectral count (Nsc)^24^.

GO enrichment analysis of proteins was conducted using DAVID Bioinformatics Resources 6.8 (https://david.ncifcrf.gov/). KEGG pathway analysis was conducted as previously described^50^. Protein-protein interaction networks were generated using STRING (http://string-db.org)^55^. Heatmaps were generated using R-package software.

### sMB-R uptake and KRAS^G12V^ transfer assay

NIH3T3 fibroblasts were grown on Nunc® Lab-Tek® Chamber Slide™ system to 70% confluency. The medium was supplemented with sMB-R (5 µg), exosomes (5 µg) or PBS vehicle and cells further cultured at 37 °C for 2 h to allow uptake. The ratio of sMB-R particles to cells are 50:1 (Fig. 4d). Cells were then subjected to immunofluorescence microscopy analysis using anti-MKLP1 or anti-KRAS^G12V^ mutant-specific antibodies.

### Soft-agar colony formation assay

NIH3T3 fibroblasts (20,000 cells) in 100 µl DMEM (1% Pen/Strep) were stimulated with 20 µg of SW620-sMB-R or 20 µl PBS vehicle for 2 h at 37 °C. The ratio of sMB-R particles to cells are 250:1 (Fig. 5b). Where indicated, experiments were performed in the presence of 10 nM selumetinib or DMSO vehicle. Fibroblasts were then mixed with 300 µl 0.3% agarose (in DMEM with 10% FBS, 1% Pen/Strep) that was pre-warmed to 40 °C in a water bath. The mixture was overlaid onto wells of a 24-well plate pre-coated with 300 µl 0.6% agarose (in DMEM with 10% FBS, 1% Pen/Strep). The mixture was allowed to solidify at 37 °C for 15 min. The wells were then gently overlaid with 500 µl DMEM (5% FBS, 1% Pen/Strep) supplemented with 10 nM selumetinib (AZD6244) or DMSO vehicle and maintained at 37 °C for 10 days. Culture medium was replaced every 2 days. Colonies were imaged using Zeiss AxioObserver Z1 microscope (Zeiss) under bright-field.

### Transwell-Matrigel^TM^ invasion assay

Transwell-Matrigel^TM^ invasion assay was performed as previously described^47^. Briefly, Transwell inserts (8 µm pore size, Corning) were coated with 100 µl of 1 mg ml^−1^ growth factor reduced Matrigel^TM^ and allowed to polymerise for 4 h at 37 °C. NIH3T3 fibroblasts (50,000 cells) in DMEM (1% Pen/Strep) were incubated with either sMB-R (30 µg ml^−1^) or PBS alone for 2 h at 37 °C. The ratio of sMB-R particles to cells are 75:1 (Fig. 5a, c, d). Where indicated, 400 nm Polystyrene latex beads (Thermo Fisher Scientific) were incubated with cells at 75:1. Cells were then carefully overlaid onto Matrigel^TM^-coated inserts. The inserts were placed into wells of 24-well plate companion plate (Corning) that contained DMEM (5% FCS, 1% Pen/Strep) supplemented with either sMB-R (30 µg ml^−1^) or PBS alone. Invasion chambers were incubated overnight (~16 h) at 37 °C to facilitate invasion. Experiments were performed in the presence of 10 nM selumetinib or DMSO vehicle, as indicated. Inserts were washed, cells fixed (4% (v/v) formaldehyde, 5 min), and nuclei stained with Hoechst stain (10 µg ml^−1^) for 20 min. Non-invading cells were removed from the upper side of the inserts using cotton swab. Nuclei of fibroblasts that invaded to the lower side of the insert were imaged using Zeiss AxioObserver Z1 microscope. Centre of the membrane was imaged for each inset. Images were quantified using Image J software v1.49e.

Depletion of sMB-Rs was performed using Biotin-Annexin V (BD Biosciences). Briefly, we incubated purified sMB-Rs (150 µg) with Biotin-Annexin V (5 µl, BD Biosciences) or biotin-IgG (5 µg per 5 µl, Sigma Aldrich) in 500 µl 10 mM Hepes (pH 7.4), 140 mM NaCl, and 2.5 mM CaCl_2_ solution for 30 min at room temperature using gentle rotation and centrifuged at 10,000 × *g* (30 min, 4 °C). The pellet was resuspended in 150 µl PBS with Dynabeads® M-280 Streptavidin (100 µg, Thermo Fisher Scientific), and incubated at room temperature for 30 min using gentle rotation. The magnetic beads were separated with a magnet for 3 min. The unbound fractions were then used for Transwell-Matrigel^TM^ invasion assay at 1:1.

### Generation of SW620-GAP-GFP cells

pE-Growth-associated protein (GAP) (1-20 a.a., MLCCMRRTKQVEKNDEDQKI)-GFP plasmid was transfected using Lipofectamine^TM^ 2000 (Invitrogen) into SW620 cells that were seeded to 70% confluency in 6-well plate. Briefly, 10 µg of plasmid was mixed with 10 µl of Lipofectamine^TM^ 2000 in 500 µl RPMI medium at room temperature for 20 min. Cells were washed and overlaid with 1.5 mL RPMI (10% FBS). Plasmid-Lipofectamine^TM^ mixture was then overlaid onto the cells. Cells were incubated at 37 °C with 10% CO_2_. Cells with stable expression of GAP-GFP fusion proteins were selected following multiple rounds of single cell cloning into wells of 96-well plate. Expression of the GAP-GFP fusion protein in the expanded colonies was monitored using a Zeiss AxioObserver Z1 microscope and analysed by BD FACSCanto II HTS (BD Biosciences) using FlowJo software (TreeStar).

### Statistics and reproducibility

Quantitative data represented as mean ± standard error of mean (s.e.m.). Statistical analyses were performed using GraphPad Prism software (one-way ANOVA (Turkey test)) with *P* < 0.05 considered as statistically significant. No method of randomisation was used. Investigators were not blinded to allocation during experiments or outcome assessment.

For proteomics analysis, experiments were performed in biological duplicate (with technical duplicates). Data were searched with a parent tolerance of 10 ppm, fragment tolerance of 0.5 Da and minimum peptide length 7, with FDR 1% at the peptide and protein levels. Peptide spectral matches were validated using Percolator based on *q*-values at a 1% false discovery rate (FDR)^52^. Scaffold (Proteome Software Inc., Portland, OR, v 4.3.4) was employed to validate MS/MS-based peptide and protein identifications from database searching^54^. Initial peptide identifications were accepted if they could be established at greater than 95% probability as specified by Peptide Prophet^53^. Protein identifications were accepted, if they reached greater than 99% probability and contained at least two identified unique peptides. Protein probabilities were assigned by Protein Prophet^54^.

Fluorescence microscopy analysis of MB-R shedding was performed ≥3× for SW620 cells and 2× for SW480 cells (Fig. 1a and Supplementary Fig. 3). Fluorescence microscopy analysis of MB-R shedding by SW620, SW480, LIM1215 (Fig. 1c) and mouse intestinal organoids (Supplementary Fig. 8) was performed 3×. Shedding of MB-R in vivo was analysed in 4 SW620-GAP-GFP tumour xenografts (Fig. 1d). Cryo-EM imaging in Fig. 2e was performed ≥3× for Exos, 3× for sMV-LD and 2× for sMB-R with similar results. Numbers of EVs counted from Cryo-EM analysis in Fig. 2f were 60 for exos, 50 for sMV-LD and 30 for sMB-R. NTA analysis of three EV subtypes in Supplementary Fig. 13 was performed ≥3×. Immunofluorescence detection of sMB-R on aldehyde sulphate latex beads (Fig. 2g) was performed 2×. Co-IP experiment was performed 2×. Relative protein abundance of three EV subtype was performed in duplicate from five different cell lines (Fig. 2h).

Western blot-based validation of specific proteins for each EV-subtype was performed 2× for Fig. 2d, Supplementary Figs. 9, 12, and 15. Western blot analysis for Fig. S10 was performed 1×. Isolation of three EV subtypes from CM was performed ≥3× for SW620, SW480 and LIM1863 cells and 2× for MBA MB 231 and U87 cells. Western blot analysis of MB-R shedding into CM by multiple cell types in Fig. S16 was performed 1×. Western blot analysis of Supplementary Fig. 10 was performed 1×.

Immunofluorescence microscopy analysis of KRAS^G12V^ in SW620 MB/ MB-R (Fig. 3f) was performed 3×. Western blot detection of KRAS^G12V^ in SW620 sMB-R (Fig. 3g) was performed 3×. Uptake of sMB-Rs by NIH3T3 in Fig. 4a and Fig. S19 was performed ≥3×. Confocal microscopy of sMB-R uptake and internalisation by NIH3T3 was performed 3× (Fig. 4c). Transfer of KRAS^G12V^ by sMB-R to NIH3T3 fibroblasts (Fig. 4d) was performed 2×. Anchorage independent growth assay (Fig. 5b, Supplementary Fig. 20) and Transwell invasion assays (Fig. 5a, c, d) were performed ≥3×.

For Western blot, antibodies were validated as noted on manufacturer’s website. For immuno-fluorescence, antibodies were validated as noted on manufacturer’s website. Uncropped/entire Western blot images are provided in Supplementary Fig. 22.

### Reporting summary

Further information on experimental design is available in the Nature Research Reporting Summary linked to this paper.

## Supplementary information

Supplementary Information

Description of Additional Supplementary Files

Supplementary Data 1

Supplementary Data 2

Supplementary Data 3

Supplementary Data 4

Supplementary Data 5

Supplementary Data 6

Supplementary Data 7

Supplementary Data 8

Supplementary Data 9

Reporting Summary

## Data Availability

Raw mass spectrometry data is deposited in the PeptideAtlas: #PASS01206 or can be accessed at http://www.peptideatlas.org/PASS/PASS01206 Source data for Figs. 2f, i, 4b, 5a–d, S7 and S20b have been provided as Supplementary Data 9. MKLP1- or RACGAP1-antibodies based immunohistochemical images of human colon cancer tissues are publicly available from the Human Protein Atlas (http://www.proteinatlas.org/) (Fig. 1d, Supplementary Figs. 5 and 6) and published here with permission. MiCroKITS (version 4.0) manually-curated database contains experimentally-verified midbody, centromere, kinetochore, telomere and spindle proteins^12^ (http://microkit.biocuckoo.org). Any remaining information can be obtained from the corresponding author upon reasonable request.
