# Supplementary material for: Secreted midbody remnants are a class of extracellular vesicles molecularly distinct from exosomes and microparticles
Source: Commun Biol. 2021 Mar 25;4:400. doi: 10.1038/s42003-021-01882-z (PMC7994562; doi:10.1038/s42003-021-01882-z)

SUPPLEMENTARY INFORMATION

Secreted midbody remnants are a class of extracellular vesicles molecularly distinct from exosomes and microparticles

*Alin Rai, David W Greening, Rong Xu, Maoshan Chen, Wittaya Suwakulsiri and Richard J Simpson**

Department of Biochemistry and Genetics, La Trobe Institute for Molecular Science, La Trobe University, Melbourne, Victoria, Australia.

**e-mail: richard.simpson@latrobe.edu.au*

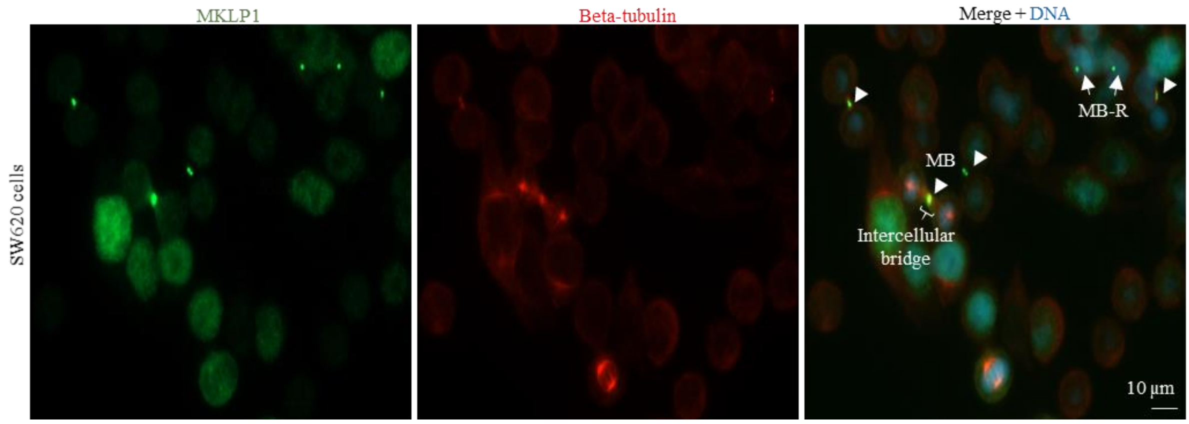

Supplementary Figure 1. Immunostaining of SW620 cells with anti-MKLP1 (in green) and anti-beta-tubulin (in red) antibodies validate MKLP1 as a marker of MB/MB-R. Prospective daughter cells are connected by the intercellular bridge stained with anti-beta-tubulin antibody and at the centre of the bridge lies the midbody (MB, white arrowheads) that is co-stained with anti-MKLP1 and anti-beta-tubulin antibodies. MKLP1-positive puncta associated with non-dividing cell represent the midbody remnant (MB-R, white arrows). Nuclei stained with Hoechst (blue).

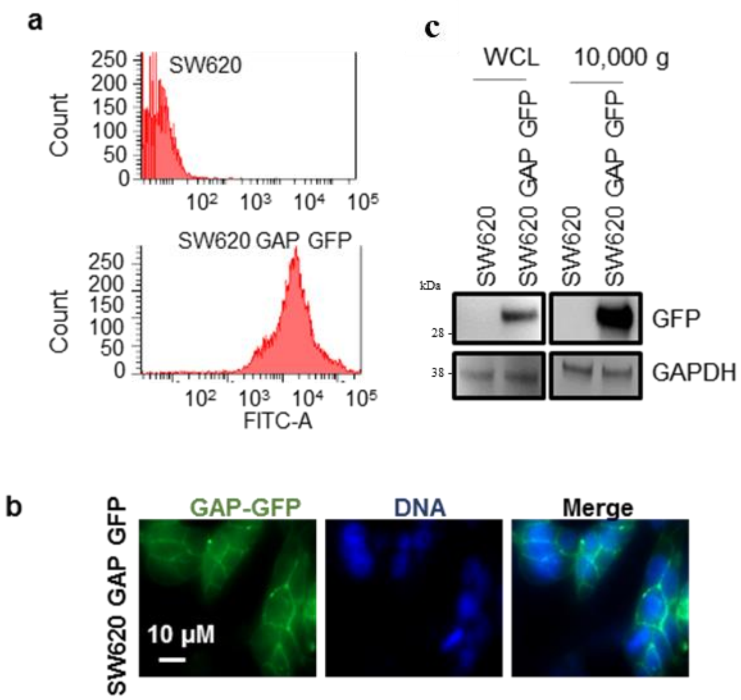

Supplementary Figure 2. Generation of SW620-GAP-GFP cell line. a, FACS analysis of SW620 stably expressing green fluorescent protein (GFP)-tagged neuronal growth-associated protein (GAP-43), abbreviated here as GAP-GFP protein. b, Fluorescent microscopy analysis of SW620 GAP-GFP cells. Nuclei are stained with Hoechst in blue. c, Western blot analysis of whole cell lysate derived from SW620 and SW620-GAP-GFP cells using anti-GFP and anti-GAPDH antibodies. Each lane was loaded with 10 μg of protein.

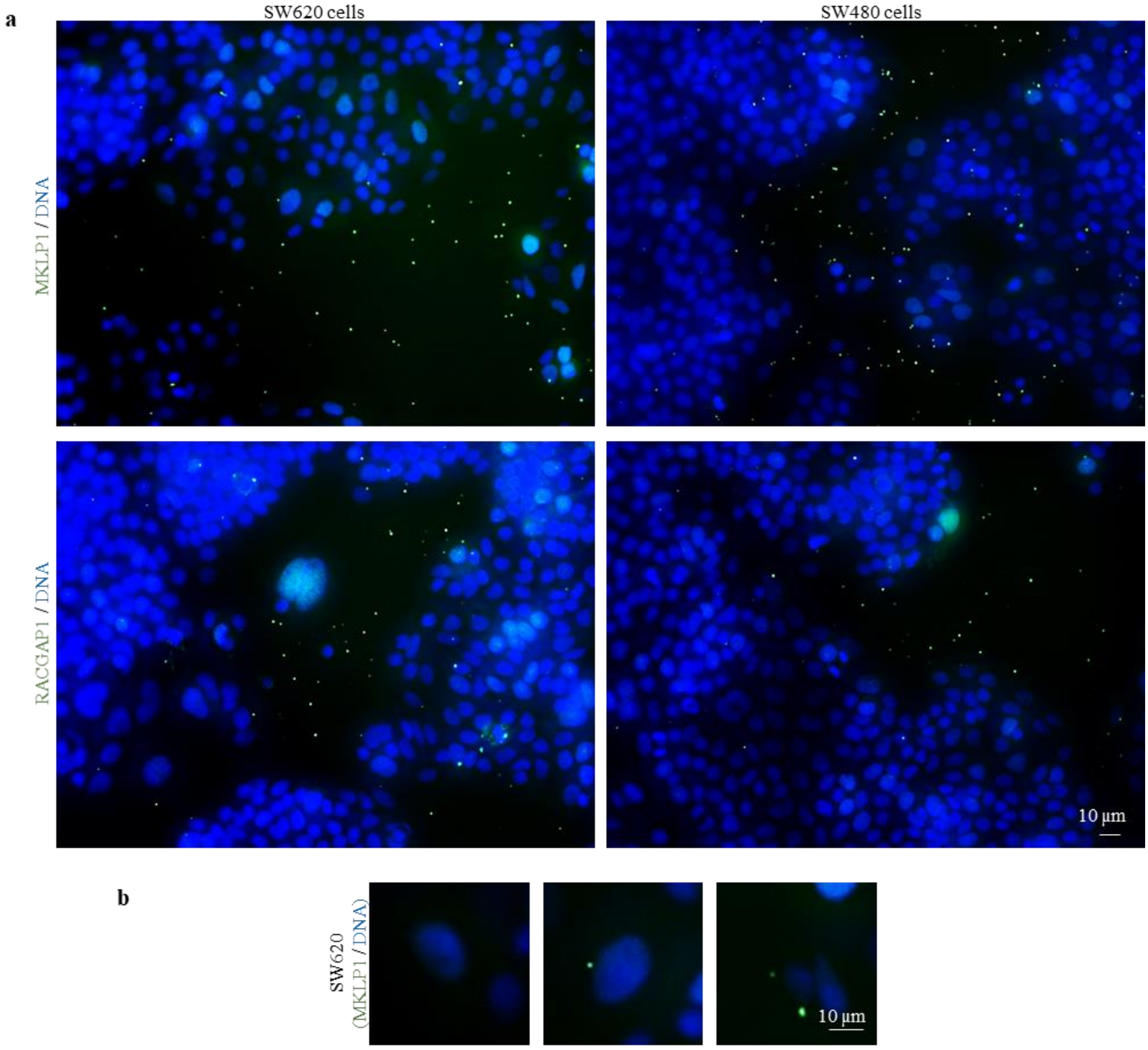

MB-R / cell	= 0	= 1	> 1
SW620 (n = 582)	88.7 %	9.7 %	1.6 %
SW480 (n = 439)	92.7 %	6.4 %	0.9 %

Supplementary Figure 3. SW480 and SW620 cells shed MB-Rs into extracellular space. a, Fluorescence microscopic analysis of SW480 or SW620 cells using anti-MKLP1 or anti-RACGAP1 antibodies (Green). Nuclei stained with Hoechst (Blue). b, A representative fluorescent microscopic image of SW620 cell with no MB-Rs or with 1 or >1 MB-R. Nuclei are stained with Hoechst (Blue). Number of MKLP1-positive MB-R puncta per cell (0, 1 or >1) were manually counted and represented as a percentage for SW620 (number of cells counted=582) and SW480 cells (number of cells counted=439).

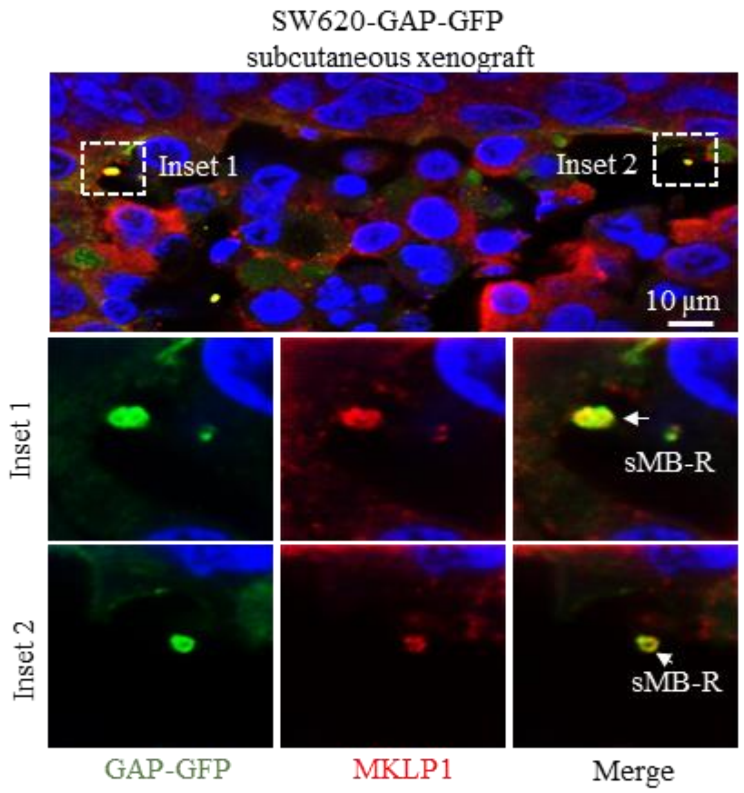

Supplementary Figure 4. Confocal fluorescence microscopy analysis of SW620-GAP-GFP subcutaneous tumours in mice using anti-MKLP1 antibody (red). Insets: higher magnification of GFP- and MKLP1-staining sMB-R (white arrows) in the extracellular space. Scale bar, 10 µm.

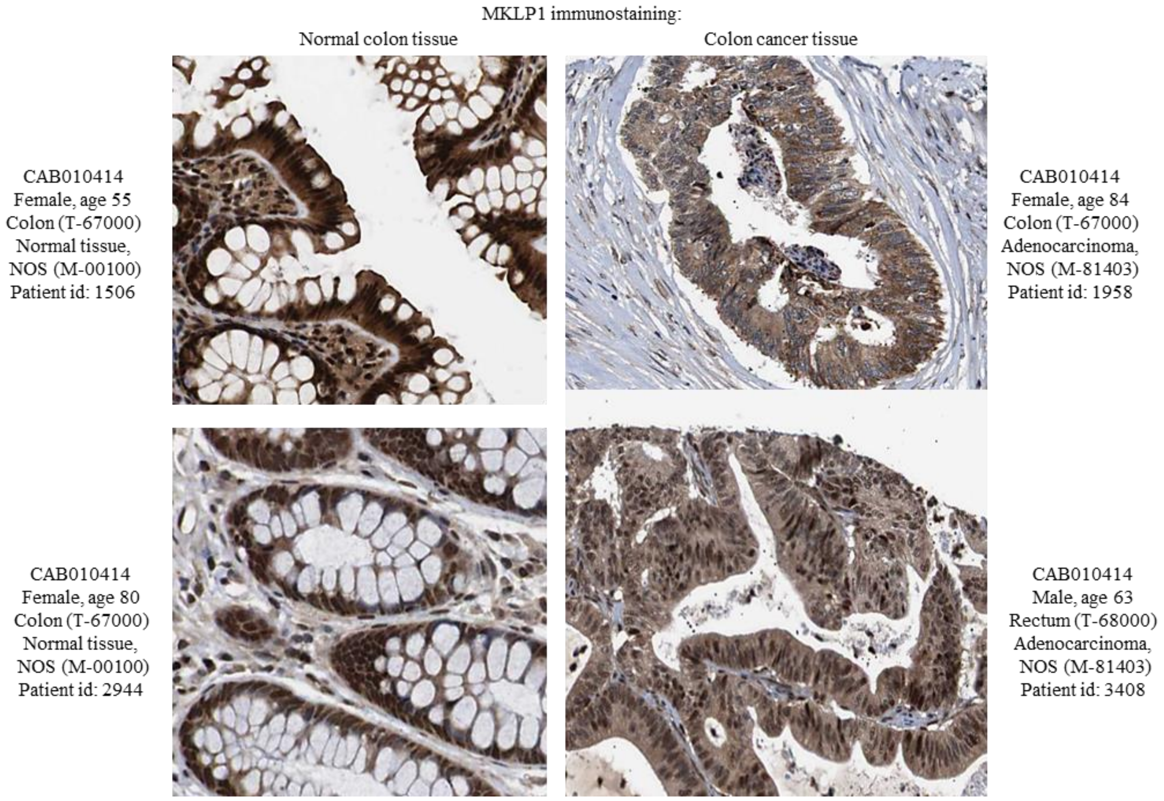

Supplementary Figure 5. Anti-MKLP1 antibody-based Immunohistochemistry analysis of normal colon tissues (n=2) and colon cancer tissue (adenocarcinoma) (n=2) publicly-available in the Human Protein Atlas (http://www.proteinatlas.org/)**.**

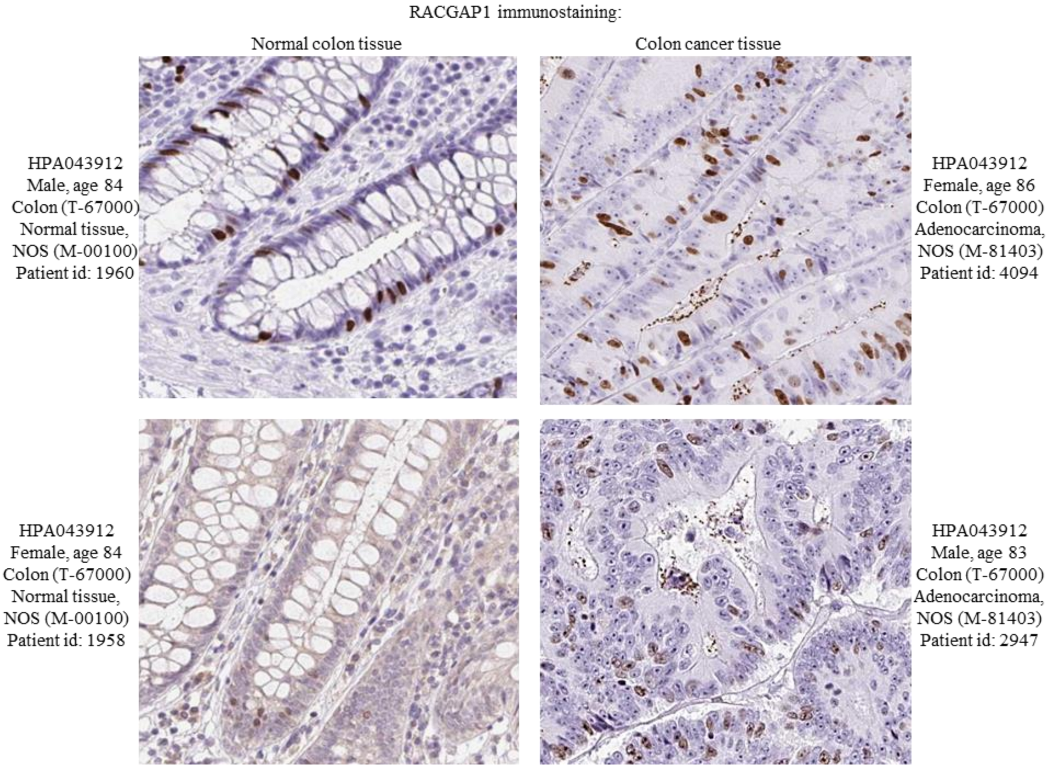

Supplementary Figure 6. Anti-RACGAP1 antibody-based Immunohistochemistry analysis of normal colon tissues (n=2) and colon cancer tissue (adenocarcinoma) (n=2) publicly-available in the Human Protein Atlas (http://www.proteinatlas.org/).

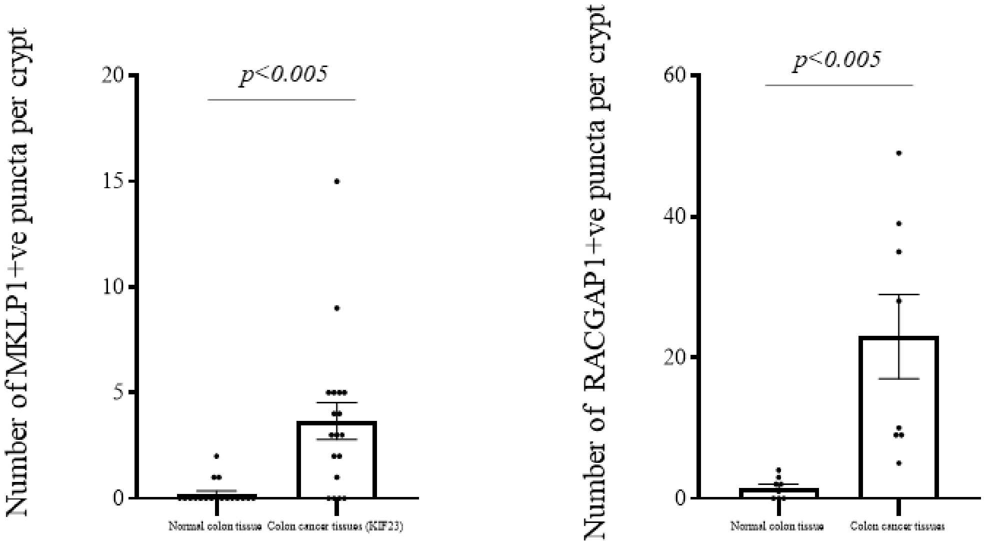

Supplementary Figure 7. Histogram of number of MKLP1 or RACGAP1^{+ve} puncta detected in normal colon tissues and colon cancer tissue (adenocarcinoma) from Supplementary Figure 5 and S6. Data represented as mean ± s.e.m

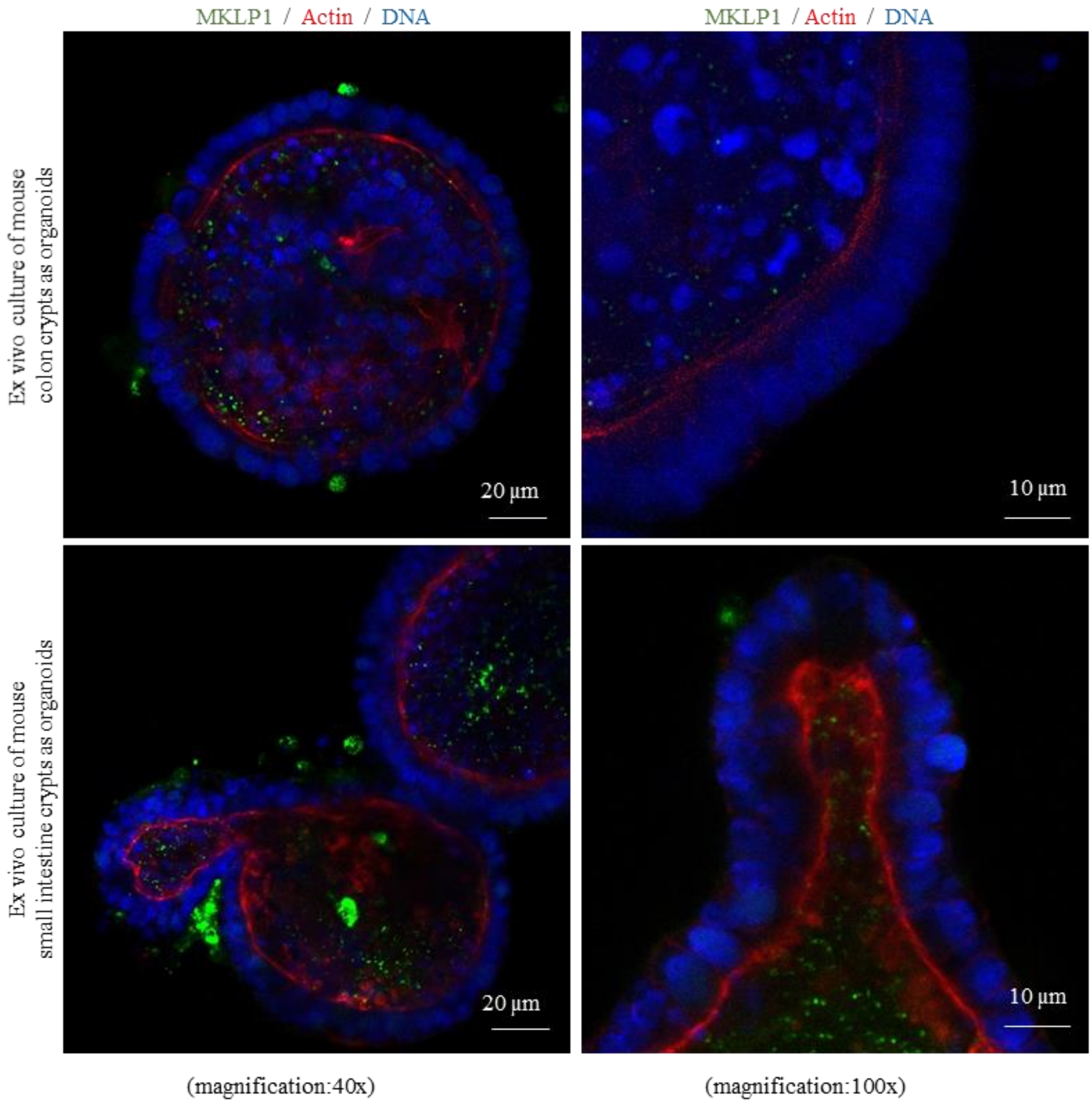

Supplementary Figure 8. Colonic or small intestinal crypts isolated from mice were cultured *ex vivo* in Matrigel™ matrix as organoids. These organoids were immunostained with anti-MKLP1 antibody (Green) and Alexa Fluor 594 Phalloidin (Red) to stain actin. Nuclei are stained with Hoechst in blue. MB-R were shed into the central lumen. Representative images at 40x and 100x magnification are presented.

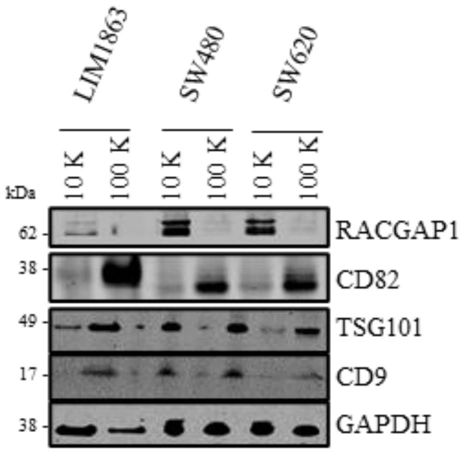

Supplementary Figure 9. Western blot analysis of crude sMVs (10,000*g* EV pellet as 10K) and crude exosomes (100,000*g* EV pellet depleted of 10,000*g* EVs as 100K) released by different cell lines using indicated antibodies

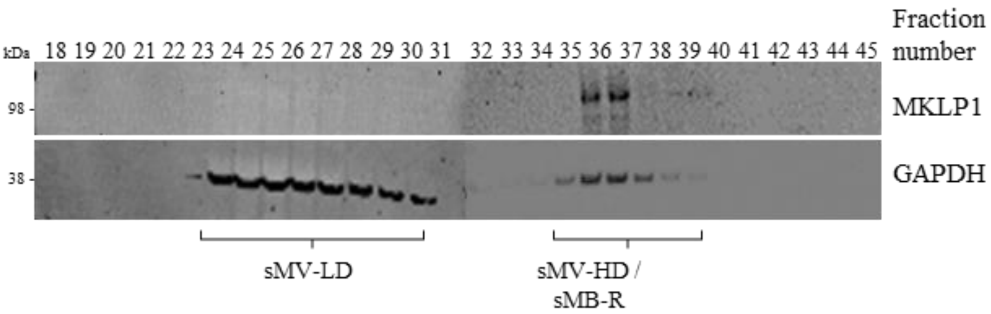

Supplementary Figure 10. Western blot analysis of 50 fractions obtained following isopycnic (iodixanol-density) centrifugation of SW620 10K demonstrates well-separated sMV-LD and sMB-Rs.

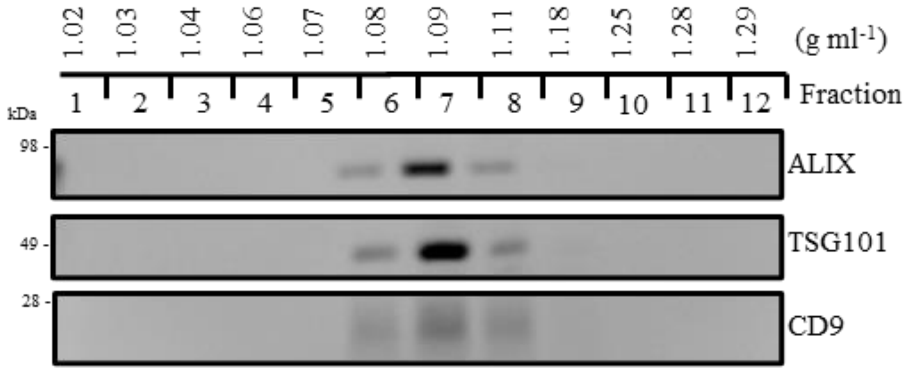

Supplementary Figure 11. Western blot analysis of 12 fractions obtained following isopycnic (iodixanol-density) centrifugation of SW620 exosomes using anti-ALIX, TSG101 and CD9 antibodies.

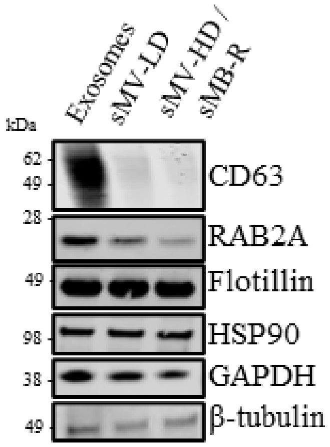

Supplementary Figure 12. Western blot analysis of Exos, sMV-LD (fractions 7-8) and sMB-R (fractions 9-10) secreted by SW620 cells using indicated antibodies.

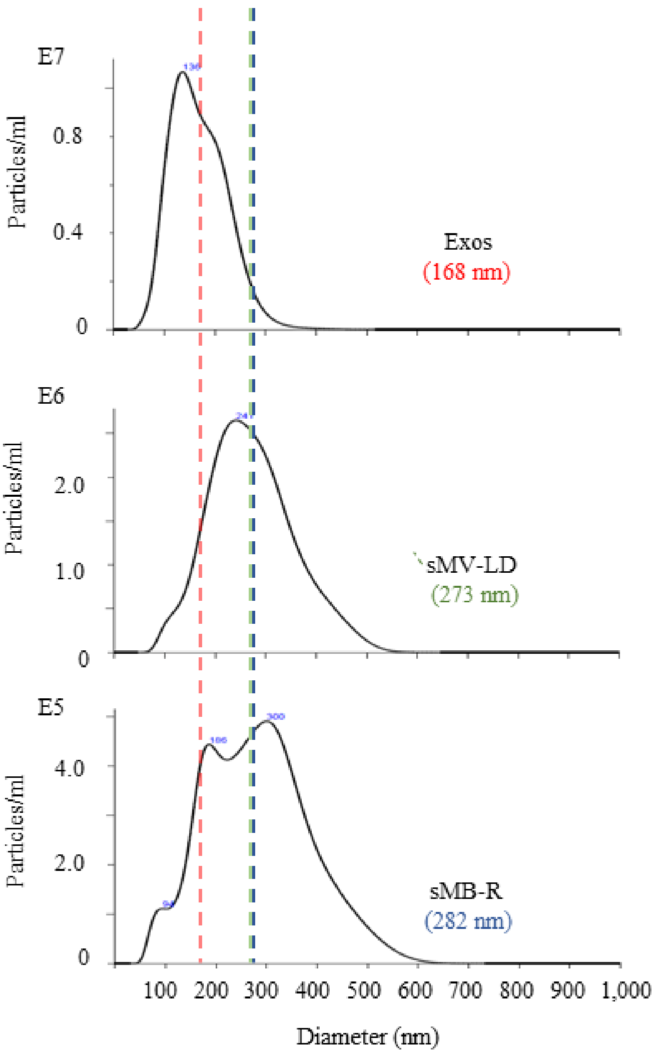

Supplementary Figure 13. Representative NTA analysis of Exos, sMV-LD and sMB-Rs derived from SW620 cells. Experiments were repeated ≥5 times with similar results. Mean diameter for each EV class is indicated.

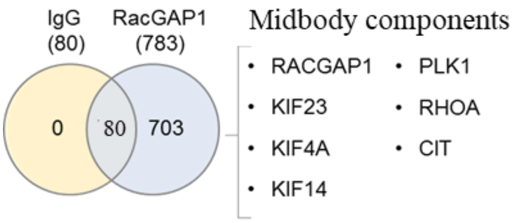

Supplementary Figure 14. Venn diagram of MS-MS identified proteins in IP of sMB-Rs from OptiPrep™ sMV-HD fractions using anti-RACGAP1 antibodies or isotype-matched IgG antibodies. Midbody component proteins identified are listed. MS-MS based identification data analysis is shown in Supplementary Data 2.

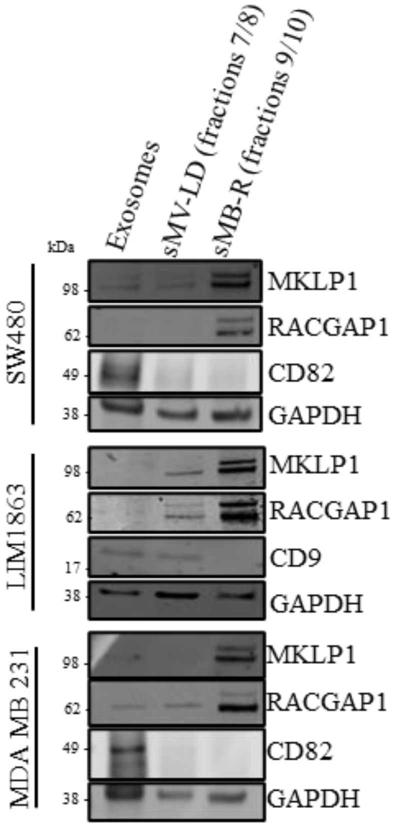

Supplementary Figure 15. Western blot analysis of exosomes, sMV-LD and sMB-Rs released by different cell lines using indicated antibodies

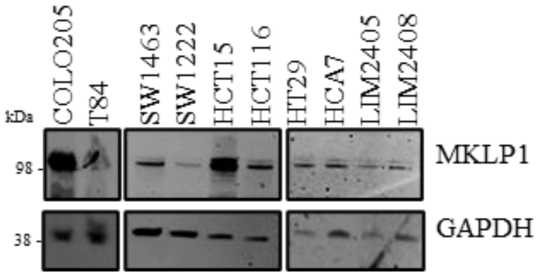

Supplementary Figure 16. Western blot analysis of crude sMVs (10,000*g* EV pellet) released by different cell lines using anti-MKLP and anti-GAPDH antibodies.

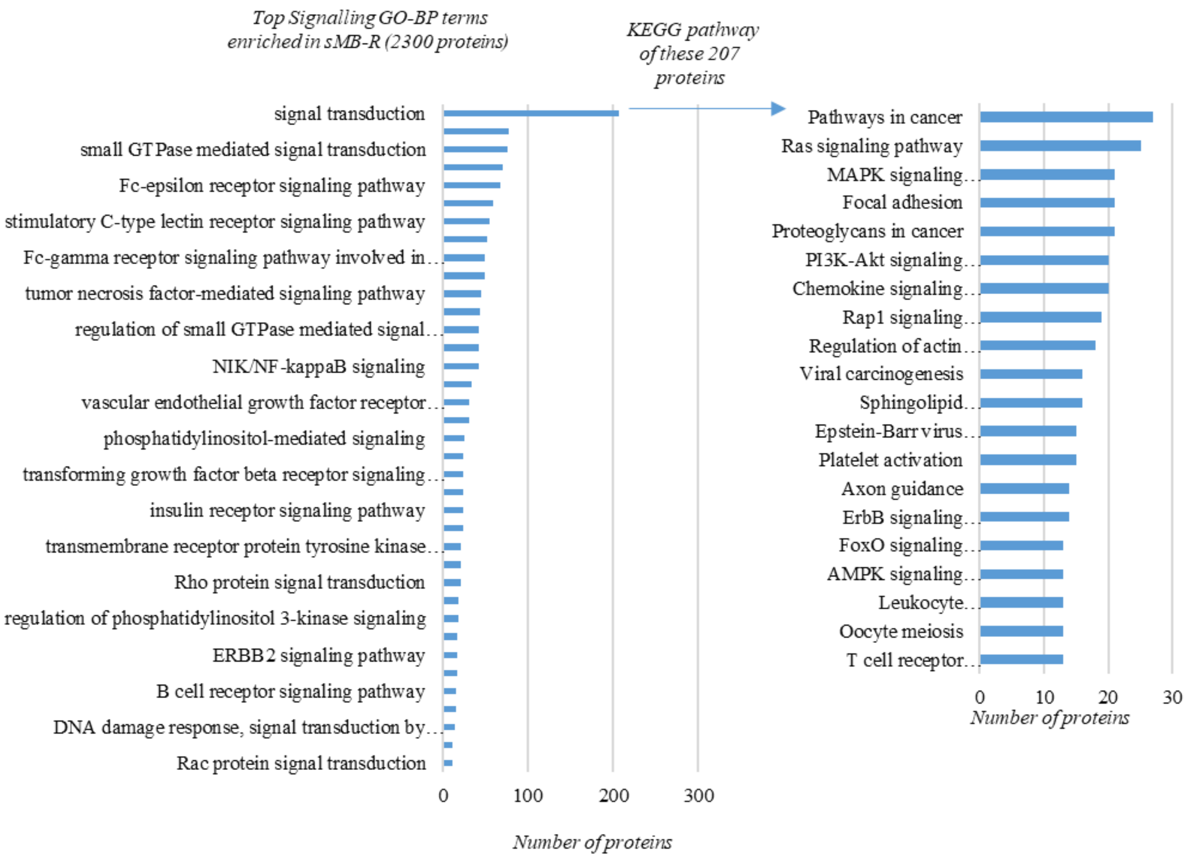

Supplementary Figure 17. DAVID (version 6.8) based Gene Ontology (GO) and Kyoto Encyclopedia of Genes and Genomes (KEGG) pathway analysis of sMB-R proteome.

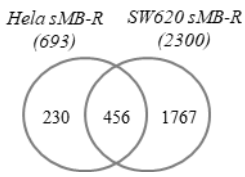

Supplementary Figure 18. Venn diagram of proteins identified in SW620 cell derived sMB-R proteome (2300 proteins) with the proteome of MB-Rs shed by Hela cells reported recently by Peterman et al. 2020. A total of 456 proteins were commonly identified (listed in Supplementary Data 6).

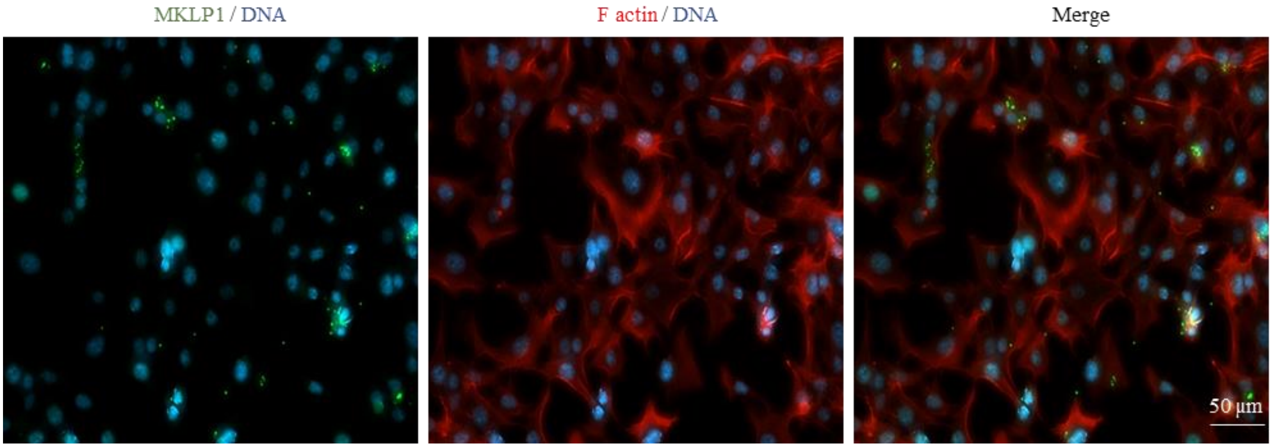

Supplementary Figure 19. Uptake of sMB-Rs by fibroblasts. Fluorescence microscopy analysis of NIH3T3 fibroblasts incubated withSW620 cell-derived sMB-Rs (50 µg/ml) for 1 h using anti-MKLP1 antibodies. F-actin was probed with Alexa Fluor™ 568 Phalloidin and nuclei (blue) were stained with Hoechst. Scale bar, 10 µm.

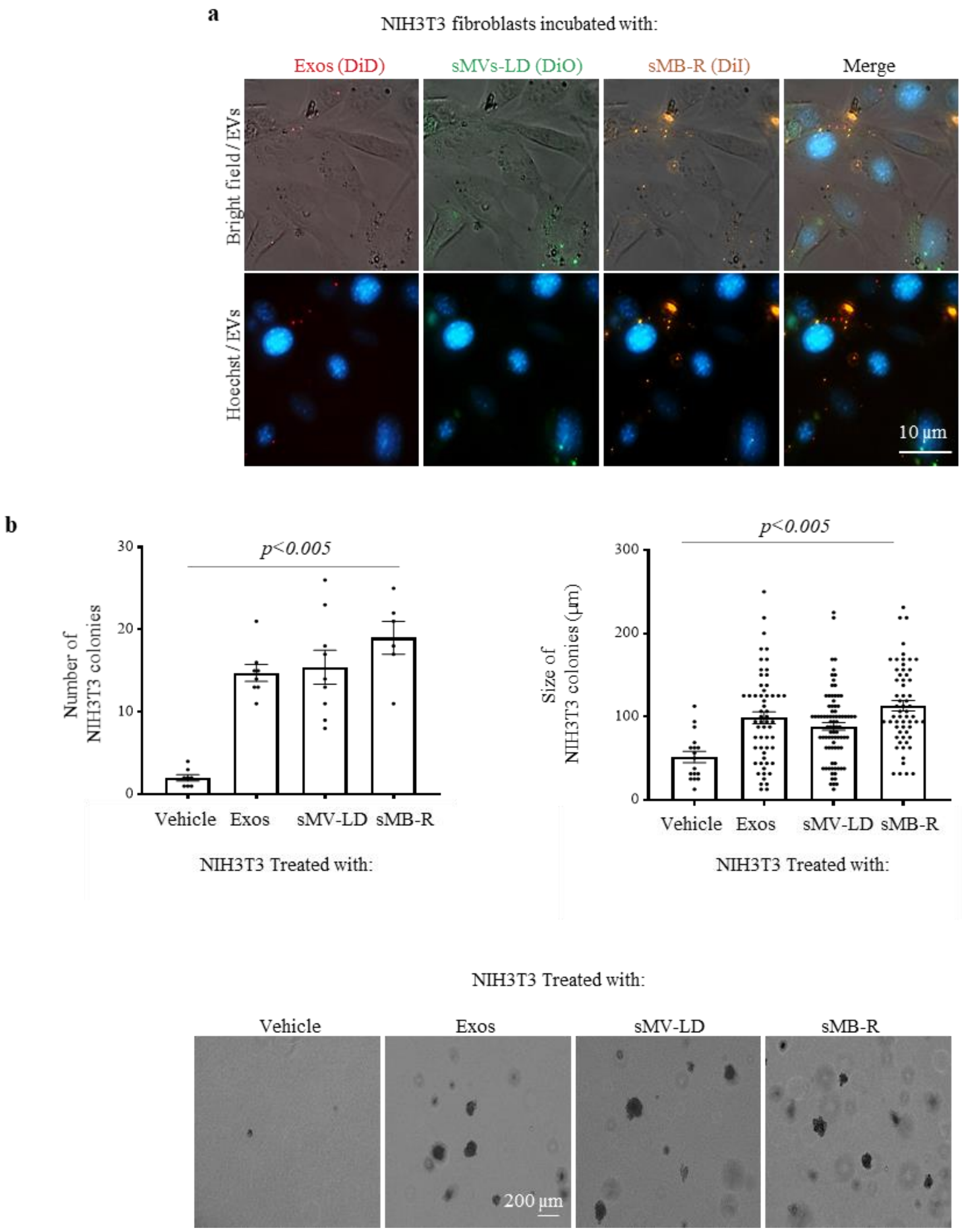

Supplementary Figure 20. a, Fluorescence microscopy analysis of NIH3T3 fibroblasts or NIH3T3 incubated with SW620 cell-derived sMB-Rs or Exos or sMV-LD for 2 h. Nuclei (blue) were stained with Hoechst. Scale bar, 10 µm. **b**, sMB-Rs or Exos or sMV-LD confer anchorage-independent growth capability in NIH3T3 fibroblasts. Soft agar colony formation assay of NIH3T3 treated with SW620-derived sMB-Rs or Exos or sMV-LD. Data represented as mean ± s.e.m. Scale bar, 200 µm.

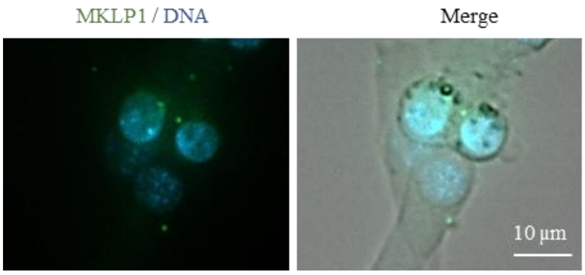

Supplementary Figure 21. Uptake of sMB-Rs by fibroblasts. Fibroblasts were treated with SW620 cell-derived sMB-Rs (50 µg/ml) for 2 h and then cultured for further 48 h. Fluorescence microscopy analysis of fibroblasts was performed using anti-MKLP1 antibodies. Nuclei (blue) were stained with Hoechst. Scale bar, 10 µm.

Supplementary Figure 22. Full western blots for indicated figures.

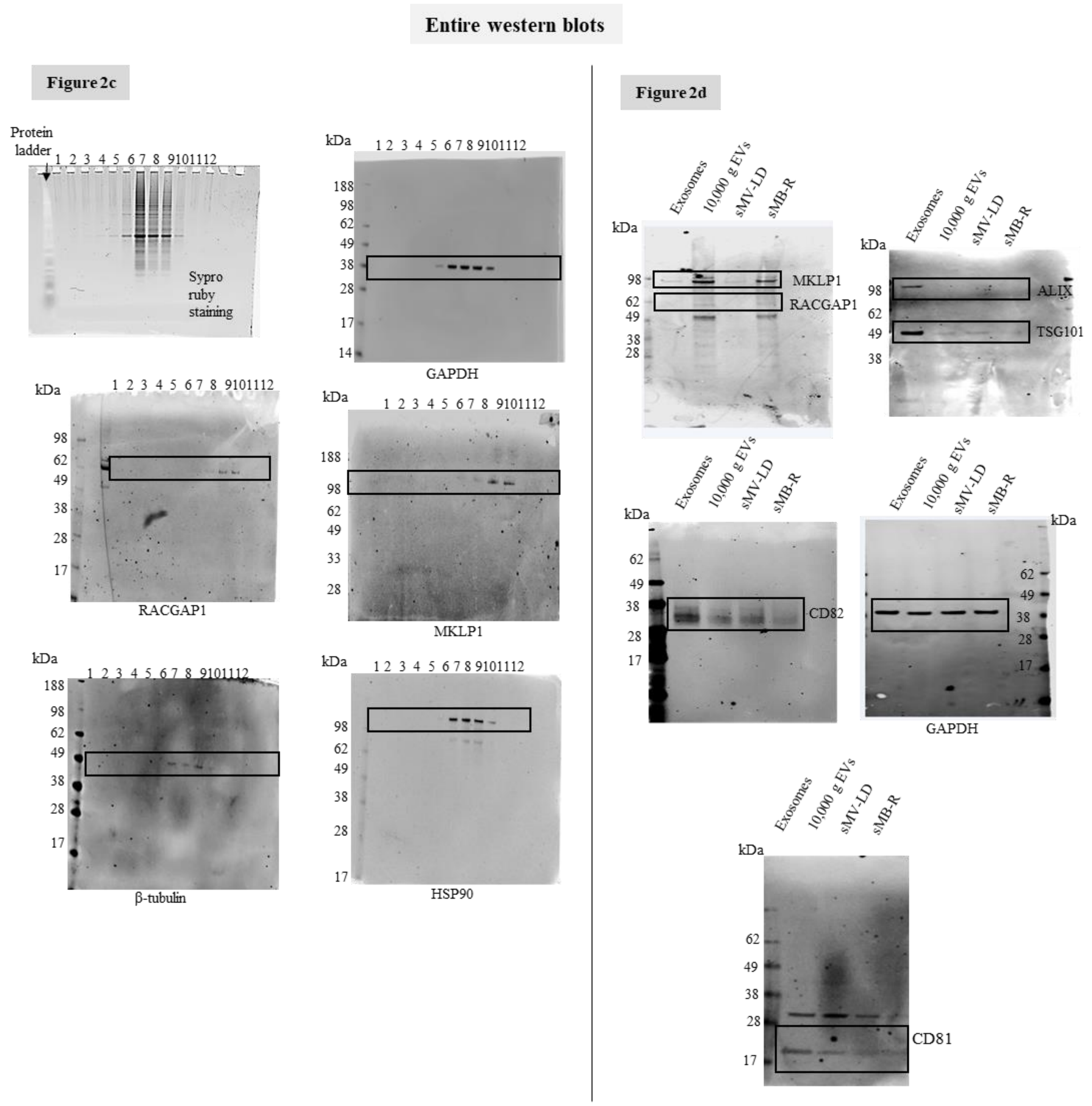

Entire western blots

Figure 3g

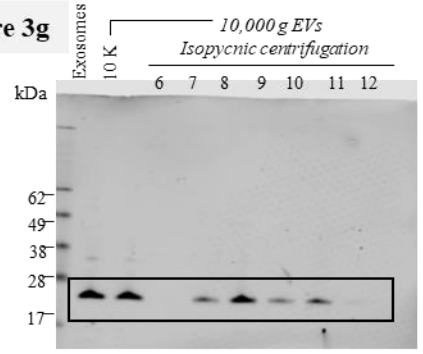

KRAS(G12V)

Figure S9

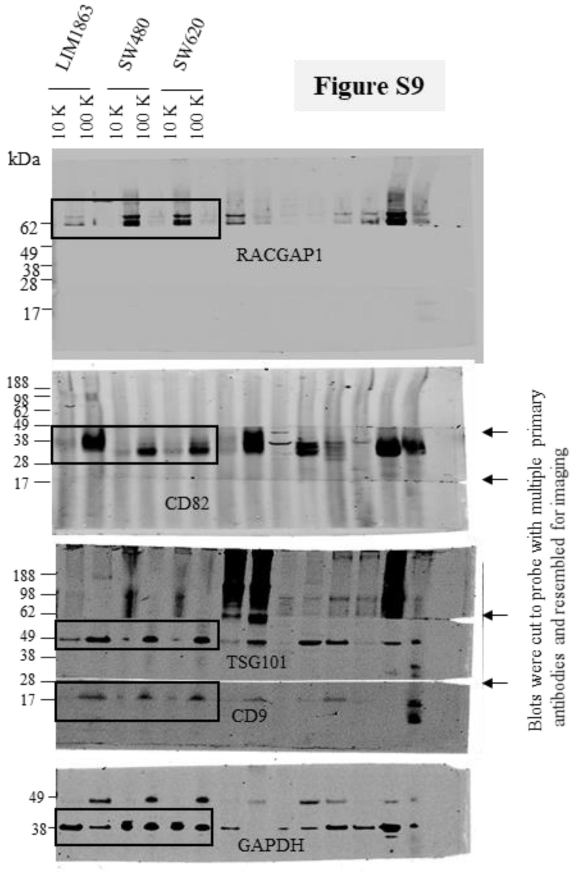

Entire western blots

Figure S10

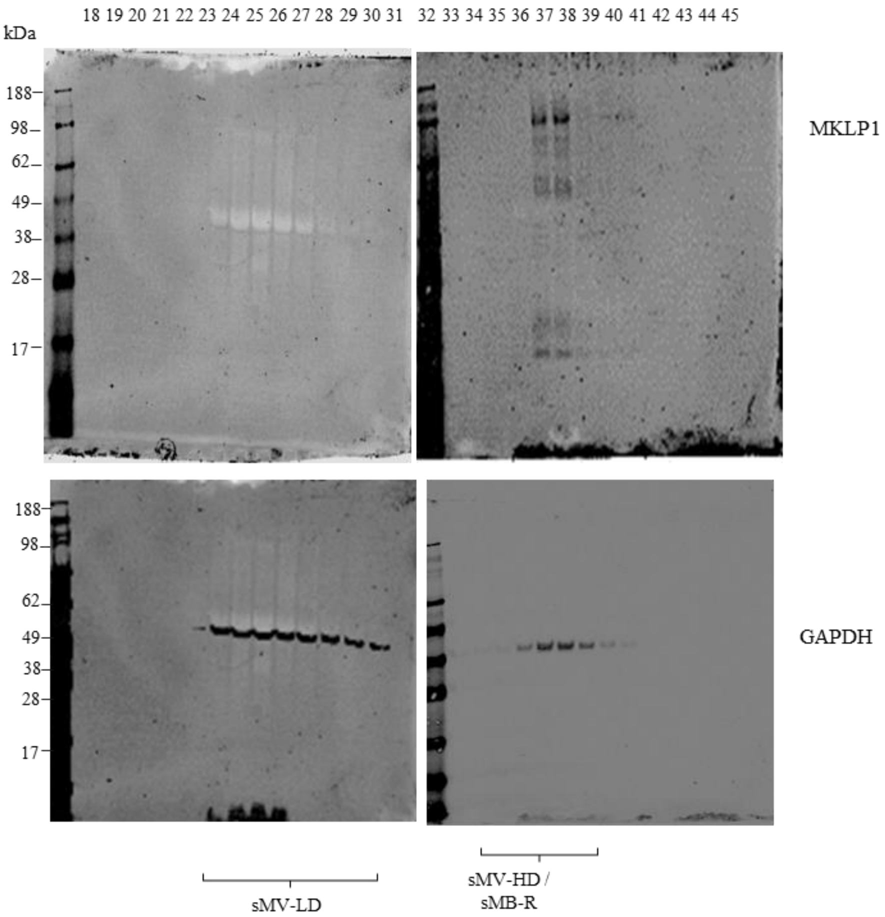

Entire western blots

Figure S11

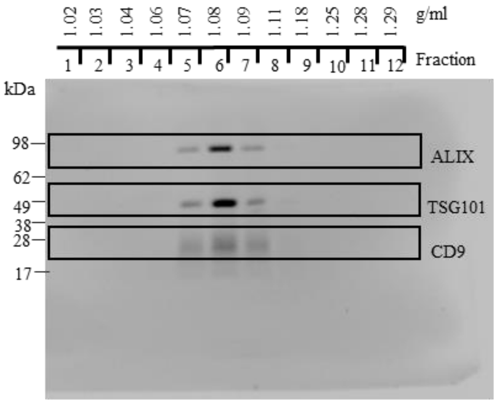

Figure S12

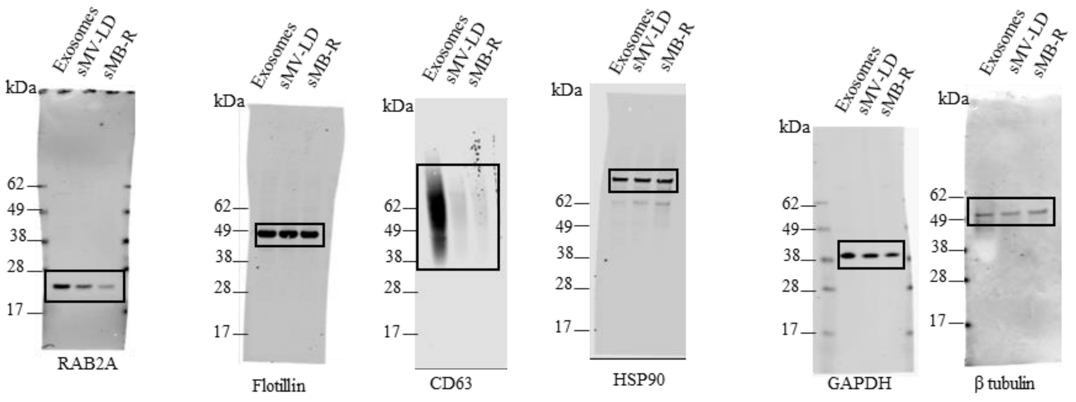

Entire western blots

Figure S15

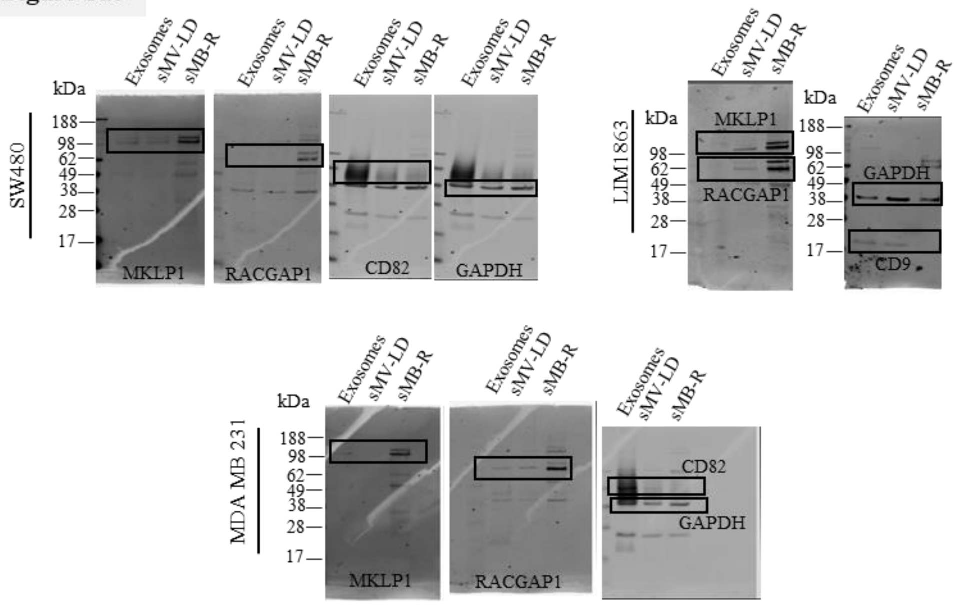

Figure S16

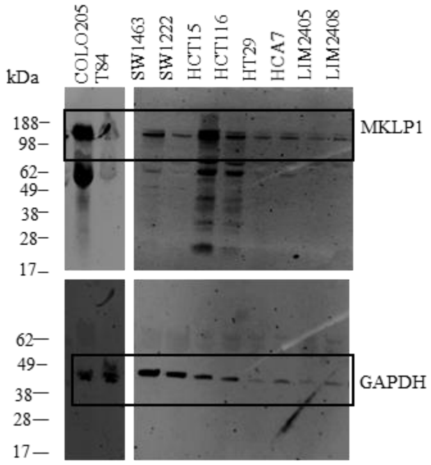

Supplement: Supplementary file 1 — Supplementary Information [file 42003_2021_1882_MOESM1_ESM.pdf]
