# Supplementary material for: Secreted midbody remnants are a class of extracellular vesicles molecularly distinct from exosomes and microparticles
Source: Commun Biol. 2021 Mar 25;4:400. doi: 10.1038/s42003-021-01882-z (PMC7994562; doi:10.1038/s42003-021-01882-z)
Supplement: Supplementary file 2 — Description of Additional Supplementary Files [file 42003_2021_1882_MOESM2_ESM.pdf]

Description of Additional Supplementary Files

File name: Supplementary Data 1

Description: Protein datasets of SW620 cell-derived exosomes, sMV-LD and sMB-Rs.

File name: Supplementary Data 2

Description: List of MS-MS-based proteins identified in RACGAP1 immune precipitate of sMB-Rs.

File name: Supplementary Data 3

Description: List of 982 SW620 cellular proteins present in high abundance in sMB-Rs, compared to exosomes and sMV-LD (highlighted in red in the heatmap shown in Fig. 3b).

File name: Supplementary Data 4

Description: List of 33 of our 982 (listed in Supplementary Data 1) identified sMB-R proteins that have been reported in Microkit database to temporally and spatially localize to distinct subcellular positions including the midbody, centrosome, kinetochore, telomere and mitotic spindle during cell division/ mitosis.

File name: Supplementary Data 5

Description: DAVID analysis of 2300 proteins identified in sMB-R.

File name: Supplementary Data 6

Description: List of 456 proteins commonly identified in SW620 cell derived sMB-R proteome (2300 proteins) and MB-Rs shed by Hela cells reported by Peterman et al. 2020.

File name: Supplementary Data 7

Description: Reactome pathways analysis of 456 proteins commonly identified in SW620 cell derived sMB-R proteome (2300 proteins) and MB-Rs shed by Hela cells reported by Peterman et al. 2020.

File name: Supplementary Data 8

Description: A complete list of proteins involved in MAPK signalling pathway identified in Reactome pathway based analysis of 456 proteins.

File name: Supplementary Data 9

Description: Statistical source data. Source data for bar plots.
