# Supplementary material for: Secreted midbody remnants are a class of extracellular vesicles molecularly distinct from exosomes and microparticles
Source: Commun Biol. 2021 Mar 25;4:400. doi: 10.1038/s42003-021-01882-z (PMC7994562; doi:10.1038/s42003-021-01882-z)
Supplement: Supplementary file 12 — Reporting Summary [file 42003_2021_1882_MOESM12_ESM.pdf]

nature research

Corresponding author(s): Alin Rai, Richard J Simpson

Reporting Summary

Nature Research wishes to improve the reproducibility of the work that we publish. This form provides structure for consistency and transparency in reporting. For further information on Nature Research policies, see Authors & Referees and the Editorial Policy Checklist.

Statistical parameters

When statistical analyses are reported, confirm that the following items are present in the relevant location (e.g. figure legend, table legend, main text, or Methods section).

n/a	Confirmed	
	x	The exact sample size (*n*) for each experimental group/condition, given as a discrete number and unit of measurement
	x	An indication of whether measurements were taken from distinct samples or whether the same sample was measured repeatedly
	x	The statistical test(s) used AND whether they are one- or two-sided<br>*Only common tests should be described solely by name; describe more complex techniques in the Methods section.*
x		A description of all covariates tested
x		A description of any assumptions or corrections, such as tests of normality and adjustment for multiple comparisons
	x	A full description of the statistics including central tendency (e.g. means) or other basic estimates (e.g. regression coefficient) AND variation (e.g. standard deviation) or associated estimates of uncertainty (e.g. confidence intervals)
	x	For null hypothesis testing, the test statistic (e.g. *F*, *t*, *r*) with confidence intervals, effect sizes, degrees of freedom and *P* value noted<br>*Give P values as exact values whenever suitable.*
x		For Bayesian analysis, information on the choice of priors and Markov chain Monte Carlo settings
x		For hierarchical and complex designs, identification of the appropriate level for tests and full reporting of outcomes
x		Estimates of effect sizes (e.g. Cohen's *d*, Pearson's *r*), indicating how they were calculated
	x	Clearly defined error bars<br>*State explicitly what error bars represent (e.g. SD, SE, CI)*

Our web collection on statistics for biologists may be useful.

Software and code

Policy information about availability of computer code

Data collection	UniProt Human database comprising 71,785 entries (Jan-2018), Proteome Discoverer (v2.1, Thermo Fischer Scientific), Mascot (Matrix Science, London, UK; v2.5), Sequest (Thermo Fisher Scientific, San Jose, CA, v1.4.0.288), X! Tandem (v2010.12.01.1), The human protein atlas (https://www.proteinatlas.org/)
Data analysis	PRISM, ImageJ, NTA software 3.0 (ATA Scientific), Zen 2011 (Blue edition, Zeiss), DAVID Bioinformatics Rescourfces 6.8 (https://david.ncifcrf.gov/), STRING (http://string-db.org), R-package software, Cytoscape 3.7.1

For manuscripts utilizing custom algorithms or software that are central to the research but not yet described in published literature, software must be made available to editors/reviewers upon request. We strongly encourage code deposition in a community repository (e.g. GitHub). See the Nature Research guidelines for submitting code & software for further information.

Data

Policy information about availability of data

All manuscripts must include a data availability statement. This statement should provide the following information, where applicable:

- Accession codes, unique identifiers, or web links for publicly available datasets
- A list of figures that have associated raw data
- A description of any restrictions on data availability

Raw mass spectrometry data is deposited in the PeptideAtlas: #PASS01206 or http://www.peptideatlas.org/PASS/PASS01206.

MKLP1- or RACGAP1-antibodies based immunohistochemical images of human colon cancer tissues are publicly available from the Human Protein Atlas (http://www.proteinatlas.org/) (Figure 1d, Supplementary Figures 5-6) and published here with permission. MiCroKITS (version 4.0) manually-curated database contains experimentally-verified midbody, centromere, kinetochore, telomere and spindle proteins12 (http://microkit.biocuckoo.org). Source data for Fig. 2f, 2i, 4b, 5a-d, S7 and S20b have been provided as Supplementary Data 9.

Any remaining information can be obtained from the corresponding author upon reasonable request.

Field-specific reporting

Please select the best fit for your research. If you are not sure, read the appropriate sections before making your selection.

[x] Life sciences   [ ] Behavioural & social sciences   [ ] Ecological, evolutionary & environmental sciences

For a reference copy of the document with all sections, see nature.com/authors/policies/ReportingSummary-flat.pdf

Life sciences study design

All studies must disclose on these points even when the disclosure is negative.

Sample size	No statistical method was used to predetermine sample size. Release of midbdoy remnant was demonstrated in multiple cell lines. Results obtained were highly significant and consistent.
Data exclusions	No data were excluded from the analyses
Replication	All attempts at replication were successful
Randomization	Samples were not randomized for the experiments
Blinding	Investigators were not blinded to group allocation during data collection and/or analysis

Reporting for specific materials, systems and methods

Materials & experimental systems

n/a	Involved in the study	
x		Unique biological materials
	x	Antibodies
	x	Eukaryotic cell lines
x		Palaeontology
	x	Animals and other organisms
x		Human research participants

Methods

n/a	Involved in the study	
x		ChIP-seq
x		Flow cytometry
x		MRI-based neuroimaging

Antibodies

Antibodies used	Rabbit antibodies against GAPDH (Cell Signalling), β-tubulin (Cell Signalling), RASG12V mutant specific (Cell Signalling), RAB7 (Abcam) and GFP (Abcam) were used. Mouse antibodies against MKLP1 (Santa Cruz), RACGAP1 (Santa Cruz), ALIX (BD Biosciences), TSG101 (BD Biosciences), CD63 (Santa Cruz), CD81 (Santa Cruz), RAB2A (Thermo Fisher), FLOT1 (BD Biosciences), α-actinin (Abcam), CD9 (Santa Cruz) and HSP90 (BD Biosciences) were used. Secondary antibodies used were IRDye 800 goat anti-mouse IgG or IRDye 700 goat anti-rabbit IgG (1:15000, LI-COR Biosciences).
Validation	For Western blot, antibodies were validated as noted on manufacturer's website.<br>In addition, their specificity was confirmed in the literature. We also tested the validation of KRAS (G12V) mutant-specific antibody by using positive and negative cells.<br><br>For immuno-fluorescence, antibodies were validated as noted on manufacturer's<br>website, and most of antibodies specificity was confirmed in the literature. In addition, the stainings were consistent with the predicted cellular localization of the protein.

Eukaryotic cell lines

Policy information about cell lines

Cell line source(s)	SW620 (CCL-227, ATCC), SW480 (CCL-228, ATCC), LIM1863 cells (The Ludwig Institute for Cancer Research, Melbourne), COLO 205 (CCL-222™, ATCC), T84 (CCL-248, ATCC), SW1463 (CCL-234, ATCC), SW1222 (12022910, CellBank Australia), LIM2405 (12062003, Sigma Aldrich), LIM2408 (The Ludwig Institute for Cancer Research, Melbourne), NIH3T3 fibroblasts (CRL-1658, ATCC), MDA MB 231 (HTB-26, ATCC), U87 (HTB-14, ATCC), HCT15 (CCL-225, ATCC), HCT116 (CCL-247, ATCC), HT29 (HTB-38, ATCC) and HCA7 (06061902, CellBank Australia) were used.
Authentication	Cell line model validation was carried out for SW480 and SW620 cells (and are also reported elsewhere). Validation data can be provided upon request.
Mycoplasma contamination	Cell lines were not tested for mycoplasma contamination.
Commonly misidentified lines<br>(See ICLAC register)	*Name any commonly misidentified cell lines used in the study and provide a rationale for their use.*

Animals and other organisms

Policy information about studies involving animals; ARRIVE guidelines recommended for reporting animal research

Laboratory animals	5-6 weeks old immunodefficient (NOD-scid) female mice were used in our study for SW620-GAP-GFP tumor xenograft establishment. Intestinal crypts were isolated from small intestine or colon of C57BL/6 mice.
Wild animals	n/a
Field-collected samples	n/a
